# Cardiac Biomarker Levels and Their Prognostic Values in COVID-19 Patients With or Without Concomitant Cardiac Disease

**DOI:** 10.3389/fcvm.2020.599096

**Published:** 2021-01-20

**Authors:** Jia-Sheng Yu, Nan-Nan Pan, Ru-Dong Chen, Ling-Cheng Zeng, Hong-Kuan Yang, Hua Li

**Affiliations:** ^1^Department of Neurosurgery, Tongji Hospital, Tongji Medical College, Huazhong University of Science and Technology, Wuhan, China; ^2^Department of Nephrology, Wuhan Fourth Hospital, Wuhan, China; ^3^Puai Hospital, Tongji Medical College, Huazhong University of Science and Technology, Wuhan, China

**Keywords:** COVID-19, cardiac biomarkers, prognostic value, cardiac disease, in-hospital mortality

## Abstract

The coronavirus disease 2019 (COVID-19) pandemic has become a global threat. Increases in cardiac biomarkers are common and are associated with adverse outcomes in patients with COVID-19. Although these increases are more likely to occur in cases with concomitant cardiac disease, the differences in cardiac biomarker levels between patients with and without cardiac disease and their associations with in-hospital mortality are largely unknown. A consecutive serial of laboratory-confirmed COVID-19 cases was retrospectively enrolled. Clinical characteristics, laboratory results, and outcome data were collected. The levels of cardiac biomarkers were evaluated and compared by stratifying patients according to concomitant cardiac conditions and clinical classifications. The prognostic efficacy of cardiac biomarker levels on admission was also assessed. Among the overall study population and survived patients, the cardiac biomarker levels at both the early and late stages in cardiac patients were significantly higher than those in non-cardiac patients. However, their concentrations in cardiac patients were comparable to non-cardiac ones among non-survivors. The cardiac biomarker levels at the late stage of the disease were significantly decreased compared to those at the early stage among patients who were alive. Whereas, the late-stage biomarker levels were significantly increased in patients who ultimately died. Subgroup analysis illustrated that increases in cardiac biomarkers were closely related to the severity of the disease, and were prognostic for high risks of in-hospital mortality in non-cardiac, rather than in cardiac patients. Myo and NT-proBNP, rather than Hs-TnI and CK-MB, were independently associated with in-hospital mortality in the overall population and non-cardiac patients. However, these associations were not significant among cardiac patients. In conclusion, our results helped better understand the release pattern and prognostic performance of cardiac biomarkers in patients with COVID-19. Increased levels of Myo and NT-proBNP on admission could be useful markers for early identifying high-risk patients. However, special attention must be paid when implementing the prognostic function for cardiac patients.

## Introduction

The coronavirus disease 2019 (COVID-19) pandemic caused by severe acute respiratory syndrome coronavirus 2 (SARS-CoV-2) has become a global threat (1). In the absence of specific therapeutic drugs or vaccines for COVID-19, it is essential to early identify high-risk patients and take interventions accordingly. Among hospitalized patients with COVID-19, increases in cardiac biomarkers indicative of myocardial injury are common and are associated with adverse outcomes (2–6). More importantly, these increases are more likely to occur in patients with concomitant cardiac conditions, which accounts for a large proportion of COVID-19 patients (7–9). Thus, although cardiac patients seem to be susceptible to myocardial injury following SARS-CoV-2 infection, the differences in cardiac biomarker levels between cardiac and non-cardiac patients were not yet thoroughly investigated. The increased levels of cardiac biomarkers were proved to be associated with high risks of in-hospital mortality (10, 11). However, the differences regarding the prognostic performance of these biomarkers in between cardiac and non-cardiac patients were largely unknown. Therefore, this study aimed to evaluate the levels of cardiac biomarkers and to investigate their prognostic role in COVID-19 patients with or without concomitant cardiac disease. Investigating their release and prognostic values is of great significance for the early identification of high-risk patients and improving outcomes by making corresponding decisions.

## Methods

### Study Design and Participants

The study was a retrospective study conducted in the Optical Valley Campus of Tongji hospital, Tongji Medical College, Huazhong University of Science and Technology (Wuhan, China), a designated hospital for COVID-19 patients. The diagnosis of COVID-19 was according to the New Coronavirus Pneumonia Prevention and Control Program (5th edition) published by the National Health Commission of China and World Health Organization interim guidance and confirmed by RNA detection of the SARS-CoV-2 in the onsite clinical laboratory. The study was approved by the ethics committee of Tongji hospital, Huazhong University of Science and Technology (institutional review board ID: TJ-C20201102). Written informed consent was waived owing to the use of de-identified retrospective data.

For the analysis of associations among the history of heart disease, cardiac biomarkers, and the in-hospital mortality of COVID-19, the following patient characteristics at hospital admission were collected: demographic characteristics, medical history, physical examination results, and laboratory findings. Demographic characteristics obtained for the study included age, sex. Medical history included cardiac disease (including coronary artery disease, atrial fibrillation, and congestive heart failure), chronic obstructive pulmonary disease (COPD), cerebrovascular disease, cancer, chronic kidney disease, chronic liver disease, diabetes (DM), and hypertension (HP). Physical examination results included first body temperature, respiratory rate, pulse rate, systolic blood pressure (SBP), diastolic blood pressure (DBP), and saturation of pulse oxygen (SpO_2_). Laboratory findings included high sensitivity troponin I (Hs-TnI), creatine kinase-MB (CK-MB), myoglobin (Myo), N terminal pro B type natriuretic peptide (NT-proBNP), white blood cell (WBC), neutrophil (NEU), lymphocyte (LYM), and platelet counts (PLT), neutrophil and lymphocyte percentage, high sensitivity C-reactive protein (Hs-CRP), interleukin 2 receptor (IL-2R), interleukin 6 (IL-6), interleukin 8 (IL-8), tumor necrosis factor α (TNFα), D-dimer levels, fibrinogen (FIB), international normalized ratio (INR), alanine aminotransferase (ALT), aspartate transaminase (AST), albumin (ALB), globulin (GLOB), creatinine (Cr), estimated glomerular filtration rate (EGFR), glucose (GLU) and total bilirubin (TBIL).

Participants aged <18, without complete electronic medical records as mentioned above, were excluded from the study. All included patients were hospitalized at the same hospital during the same time interval.

### Clinical Classifications

According to the Guidance for Corona Virus Disease 2019: Prevention, Control, Diagnosis, and Management edited by the National Health Commission of the People's Republic of China (12), all cases were identified into four categories of mild cases, ordinary cases, severe cases, and critical cases. (1) Mild cases: the clinical symptoms are mild and no pneumonia manifestation can be found in imaging. (2) Ordinary cases: patients have symptoms like fever and respiratory tract symptoms, and pneumonia manifestation can be seen in imaging. (3) Severe cases: meeting any of the following: respiratory distress, RR ≥ 30 breaths/min; the oxygen saturation is <93% at a rest state; arterial partial pressure of oxygen (PaO_2_)/oxygen concentration (FiO_2_) ≤ 300 mmHg (1 mmHg = 0.133 kPa). Patients with > 50% lesions progression within 24–48 h in pulmonary imaging were treated as severe cases. (4) Critical cases: meeting any of the following: respiratory failure occurs and mechanical ventilation is required; shock occurs; or complicated with other organ failures that requires monitoring and treatment in ICU.

### Data Collection and Follow-Up

All clinical, laboratory, and outcome data were available and collected according to the electronic medical records using a standardized data collection form. Laboratory results of the early and late stages were collected for each participant. Data collection of laboratory results at the early stage were defined using the first-time examination at admission (within 24 h after admission) and data collection of the late-stage was defined using the last examination during hospitalization. All the laboratory data were tested in the same laboratory with the same standard. All data were checked and verified by two physicians blinded to patient identification.

To observe the risk for in-hospital mortality, all patients were followed up from admission to discharge. The follow-up data were collected from reviewing medical records by trained researchers using a double-blind method.

### Statistical Analysis

Continuous variables were presented as mean ± standard deviation or median (inter-quartile range, IQR), as appropriate. Categorical variables were presented as *n* (%). Event frequencies were compared with the chi-square test. The differences of cardiac biomarker levels between the early-stage and the late-stage groups were compared using the Wilcoxon signed-ranks test (two-tailed). Other comparisons between two groups were made with independent-samples *t*-test (normally distributed continuous variables) or Mann-Whitney *U* test (non-normally distributed continuous variables). Cumulative survival curves of in-hospital death were assessed using the Kaplan-Meier product-limit estimation method with the log-rank test. The receiver-operating characteristic (ROC) analysis was applied to determine the overall performances of cardiac biomarkers for the identification of the risk of mortality in COVID-19 patients with and without cardiac disease. The area under the ROC curve (AUC) was calculated for evaluating the performance of each biomarker. The best cut-off value was computed by the ROC curve and was calculated using the maximization of the Youden's Index. Cox proportional hazards models were used to estimate the independent effect of each biomarker for in-hospital death. Hazard ratios (HRs) and 95% confidence interval (CI) were calculated in the model. Multi-variable adjustment included age, gender, comorbidities, inflammatory and coagulation factors, which were added according to univariate analysis. Statistical analyses were performed by SPSS 22.0 (SPSS, Chicago, IL, USA), and a two-sided *p* < 0.05 was considered statistically significant.

## Results

### Participants and Baseline Characteristics of the Overall Study Population

A total of 1,284 continuously admitted patients with laboratory-confirmed COVID-19 between February 9, 2020 and March 30, 2020 were initially enrolled. Of these, 256 cases were excluded due to incomplete electronic medical records, five patients were excluded due to age limitation of the study design (age > 18 years). Of 1,023 eligible patients, 126 (12.3 %) had a history of cardiac disease. The history of coronary artery disease, atrial fibrillation, and heart failure was present in 84, 21, and 33 cases. At the time of admission, mild, ordinary, severe, and critical patients were present in 14 (1.4%), 742 (72.5%), 205 (20.0%), 62 (6.1%) cases, respectively.

To observe the risk for in-hospital mortality, all included patients were followed up from admission to discharge. The mean follow-up time was 22 days (interquartile range, 14–35). A total of 60 (5.9%) all-cause death occurred during the follow-up. 17 (13.5%) deaths occurred in the cardiac disease group and 43 (4.8%) occurred in the non-cardiac group. There is a significant difference in in-hospital mortality between the two groups (Table 1).

**Table 1:** Baseline characteristics and laboratory findings of all included patients and patients stratified based on concomitant cardiac disease.

**Characteristics**	**Total (*n* = 1023)**	**No cardiac disease (*n* = 897)**	**Cardiac disease (*n* = 126)**	***p***
Age (yrs), median (IQR)	63 (51–70)	61 (50–69)	72 (64–80)	**<0.001**
Male/Female, *n*	490/533	427/470	63/63	0.614
**COMORBIDITIES**, ***n*** **(%)**				
History of HP-*n* (%)	367 (35.9)	273 (30.4)	94 (74.6)	**<0.001**
History of DM -*n* (%)	169 (16.5)	131 (14.6)	38 (30.2)	**<0.001**
Chronic liver disease-*n* (%)	15 (1.5)	15 (1.7)	0 (0)	0.144
Stroke history-*n* (%)	48 (4.7)	37 (4.1)	11 (8.7)	**0.022**
Chronic kidney disease-*n* (%)	26 (2.5)	17 (1.9)	9 (7.1)	**<0.001**
History of COPD-*n* (%)	11 (1.1)	8 (0.9)	3 (2.4)	0.129
Cancer-*n* (%)	38 (3.7)	28 (3.1)	10 (7.9)	**0.007**
**CLINICAL CLASSIFICATIONS**, ***n*** **(%)**				
Mild cases-*n* (%)	14 (1.4)	14 (1.6)	0 (0)	0.158
Ordinary cases-*n* (%)	742 (72.5)	665 (74.1)	77 (61.1)	**0.002**
Severe cases-*n* (%)	205 (20.0)	171 (19.1)	34 (27.0)	**0.038**
Critical cases-*n* (%)	62 (6.1)	47 (5.2)	15 (11.9)	**0.003**
**PHYSICAL EXAMINATION ON ADMISSION, MEDIAN (IQR)**				
Temperature (°C)	36.5 (36.2–36.9)	36.5 (36.2–36.9)	36.5 (36.3–37.0)	0.586
Pulse (/min)	89 (80–100)	90 (80–100)	87 (78- 100)	0.112
Respire (/min)	20 (19–22)	20 (19–22)	20 (19–24)	0.113
SBP (mmHg)	133 (120–145)	132 (120–145)	137 (121–148)	0.052
DBP (mmHg)	80 (72–90)	81 (72–90)	79 (72–88)	0.253
SpO_2_ (%)	97 (95–98)	97 (95–98)	97 (95–98)	0.432
**LABORATORY TESTS ON ADMISSION, MEDIAN (IQR)**				
Hs-TnI (pg/mL)	3.1 (1.9–8.5)	2.7 (1.9–7.5)	8.7 (3.3–20.9)	**<0.001**
CK-MB (ng/mL)	0.7 (0.5–1.2)	0.7 (0.5–1.1)	1.1 (0.7–1.9)	**<0.001**
Myo (ng/mL)	36.3 (26.9–62.0)	34.9 (26.0–57.4)	53.7 (33.6–93.4)	**<0.001**
NT-proBNP (pg/mL)	89.0 (34.0–271.0)	80.0 (30.0–198.5)	352.5 (109.5–1291.5)	**<0.001**
WBC (10^∧^9/L)	5.93 (4.77–7.48)	5.91 (4.73–7.44)	6.38 (5.13–8.23)	**0.012**
NEU (10^∧^9/L)	3.77 (2.77–5.26)	3.69 (2.71–5.13)	4.61 (3.22–6.19)	**<0.001**
NEU% (%)	64.3 (55.9–74.3)	63.3 (55.6–72.9)	70.8 (61.6–82.2)	**<0.001**
LYM (10^∧^9/L)	1.32 (0.91–1.77)	1.35 (0.97–1.81)	1.09 (0.71–1.55)	**<0.001**
LYM% (%)	24.0 (15.7–31.4)	25.1 (16.6–32.0)	17.5 (10.6–26.1)	**<0.001**
Hs-CRP (mg/L)	5.8 (1.2–37.6)	4.6 (1.1–35.2)	18.8 (3.4–49.4)	**<0.001**
IL2R (U/mL)	484.0 (317.0–763.0)	462.0 (306.0–724.5)	676.5 (453.5–1032.8)	**<0.001**
IL6 (pg/mL)	3.87 (1.79–12.52)	3.59 (1.66–10.60)	10.56 (3.34–25.27)	**<0.001**
IL8 (pg/mL)	10.5 (6.7–18.3)	9.9 (6.5–18.0)	13.6 (8.6–22.3)	**<0.001**
TNFα (pg/mL)	8.3 (6.3–10.7)	8.1 (6.2–10.4)	9.7 (7.3–12.5)	**<0.001**
PLT (10^∧^9/L)	232 (181–299)	234 (184–300)	218 (165–294)	0.057
D-dimer (μg/mL FEU)	0.56 (0.24–1.40)	0.51 (0.22–1.27)	0.81 (0.42–2.50)	**<0.001**
FIB (g/L)	4.08 (3.18–5.41)	4.02 (3.14–5.39)	4.36 (3.51–5.45)	**0.042**
INR	1.05 (1.00–1.11)	1.05 (1.00–1.10)	1.07 (1.01–1.17)	**0.002**
ALT (U/L)	20.0 (13.0–36.0)	21.0 (13.0–35.0)	19.0 (14.0–38.5)	0.704
AST (U/L)	22.0 (16.0–32.0)	22.0 (16.0–32.0)	24.0 (17.0–34.0)	0.327
ALB (g/L)	37.3 (33.1–41.5)	37.6 (33.4–41.7)	35.6 (30.6–39.5)	**<0.001**
GLOB (g/L)	30.1 (26.6–34.0)	30 (26.3–34.0)	30.7 (28.2–35.2)	**0.042**
Cr (μmol/L)	67 (56–81)	67 (57–80)	69 (55–89)	0.435
EGFR (ml/min/1.73 m^∧^2)	92.8 (79.1–102.9)	93.6 (80.6–103.9)	84.9 (67.3–94.4)	**<0.001**
GLU (mmol/L)	5.60 (4.97–7.02)	5.53 (4.94–6.98)	5.96 (5.29–7.62)	**0.002**
TBIL (μmol/L)	3.89 (3.26–4.63)	3.93 (3.35–4.67)	3.34 (2.68–4.05)	**<0.001**
**Hospital stay-days, median (IQR)**	22 (14–35)	22 (13–34)	30 (18–42)	**<0.001**
**In-hospital death**, ***n*** **(%)**	60 (5.9)	43 (4.8)	17 (13.5)	**<0.001**

*p values were calculated between cardiac and non-cardiac groups by Mann-Whitney U test and chi-square test, as appropriate. IQR, interquartile range; HP, hypertension; DM, diabetes; COPD, chronic obstructive pulmonary disease; SBP, Systolic blood pressure; DBP, Diastolic blood pressure; SpO_2_, percutaneous oxygen saturation; Hs-TnI, High sensitivity troponin-I; CK-MB, creatine kinase-MB; Myo, myoglobin; NT-proBNP, N terminal pro B type natriuretic peptide; WBC, white blood cell; NEU, neutrophil; NEU%, neutrophil percentage; LYM, lymphocytes; LYM%, lymphocyte percentage; Hs-CRP, high sensitivity C-reactive protein; IL2R, interleukin 2 receptor; IL6, interleukin 6; IL8, interleukin 8; TNFα, tumor necrosis factor α; PLT, platelet; FIB, fibrinogen; INR, international normalized ratio; ALT, alanine aminotransferase; AST, aspartate transaminase; ALB, albumin; GLOB, globulin; Cr, creatinine; EGFR, estimated glomerular filtration rate; GLU, glucose; TBIL, total bilirubin. The bold p values mean that they are of significance in statistics*.

The basic clinical characteristics and laboratory findings on admission of the overall study population, and patients stratified based on concomitant cardiac disease are listed in Table 1. Compared with those cases without cardiac disease, patients with a history of cardiac disease were significantly older (72 [64-80] vs. 61 [50-69] years; *p* < 0.001) and had more concomitant hypertension, diabetes, stroke history, chronic kidney disease, and cancer (Table 1). Cardiac patients were more commonly seen in severe cases (27.0% vs. 19.1%; *p* = 0.038) and critical cases (11.9 vs. 5.2%; *p* = 0.003) in comparison with non-cardiac patients. With regard to laboratory parameters, cardiac patients had lower lymphocyte count, albumin, EGFR, and glucose levels at admission. Cardiac biomarker (Hs-TnI, CK-MB, Myo, and NT-proBNP), WBC, neutrophil count, inflammatory and coagulation factor (Hs-CRP, IL-2R, IL-6, IL-8, TNFα, D-dimer and FIB) levels were numerically higher in cardiac patients compared with non-cardiac patients (Table 1).

### Cardiac Biomarker Levels and Clinical Characteristics of Cardiac and Non-cardiac Patients Among Survivors and Non-survivors

The clinical characteristics and laboratory findings of survivors and non-survivors stratified by concomitant cardiac disease were shown in Supplementary Tables 1, 2.

Compared with survivors who had no history of cardiac disease, survivors with a history of cardiac disease were older and more likely to have concomitant hypertension, diabetes, stroke, and cancer. Moreover, they had lower levels of lymphocyte count, albumin, EGFR, and TBIL, and higher levels of SBP, cardiac biomarkers (Hs-TnI, CK-MB, Myo, and NT-proBNP), WBC, neutrophil, inflammatory factors (Hs-CRP, IL-2R, IL-6, IL-8, and TNFα) and D-dimer in comparison to survivors without cardiac disease (Supplementary Table 1).

Among 60 non-survivors, 17 had a history of cardiac disease. As shown, non-survivors with cardiac disease were more likely to have a history of hypertension and chronic kidney disease. Notably, they had significantly higher levels of SpO_2_ and platelet counts, and lower levels of CK-MB, Hs-CRP, and IL-8 on admission compared with those dying without cardiac disease, which was of opposite trends in that among the survivors (Supplementary Table 2). These findings suggest that cardiac non-survivors did not develop a more severe myocardial injury or inflammatory response than non-survivors without cardiac disease on admission.

**Figure 1:**
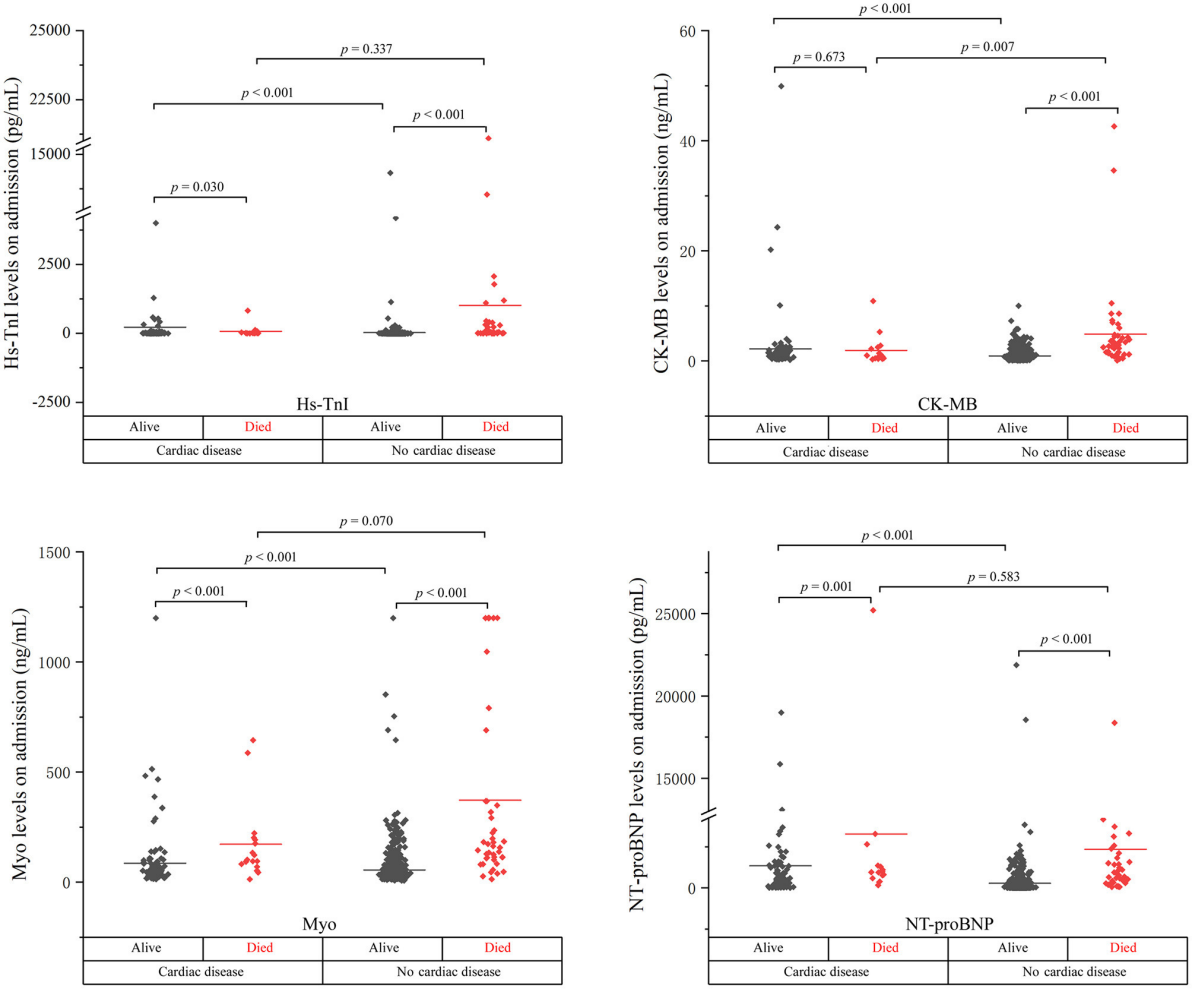
The cardiac biomarker levels at the early stage of disease in cardiac and non-cardiac patients stratified by mortality.

**Figure 2:**
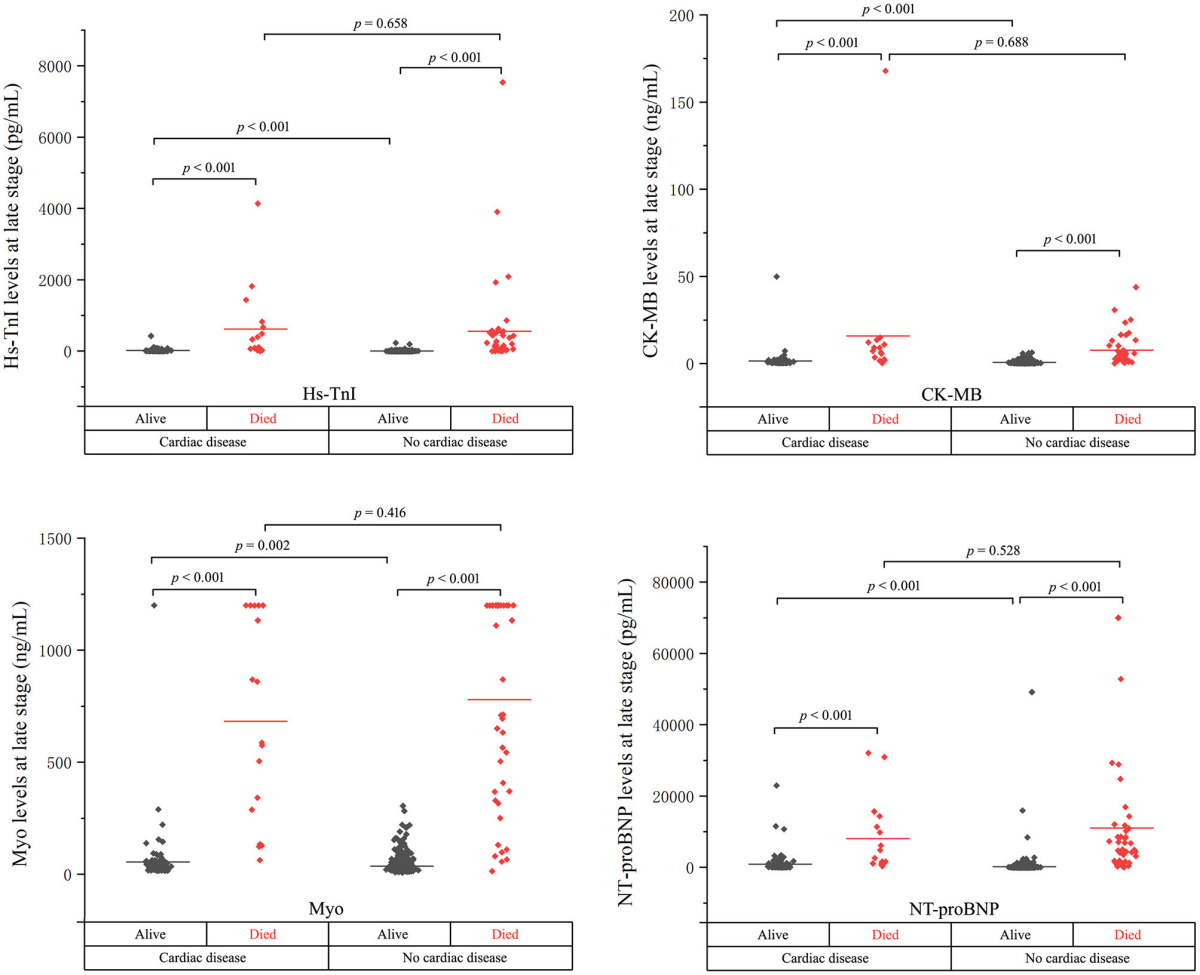
The cardiac biomarker levels at the late stage of disease in cardiac and non-cardiac patients stratified by mortality.

The levels of cardiac biomarkers in the overall study population, survivors, non-survivors, and patients stratified by concomitant cardiac disease were summarized in Table 2, Figures 1, 2.

**Table 2:** **Cardiac biomarker levels in the overall study population, survivors and non-survivors, stratified by concomitant cardiac disease**.

	**Biomarkers**	**Early stage**			**Late stage**		
		**Non-cardiac disease**	**Cardiac disease**	***p***	**Non-cardiac disease**	**Cardiac disease**	***p***
All included patients		*n* = 897	*n* = 126		*n* = 897	*n* = 126	
	Hs-TnI (pg/mL)	2.7 (1.9–7.5)	8.7 (3.3–20.9)	**<0.001**	2.1 (1.9–4.9)	6.9 (2.7–20.9)	**<0.001**
	CK-MB (ng/mL)	0.7 (0.5–1.1)	1.1 (0.7–1.9)	**<0.001**	0.6 (0.4–0.9)	1.0 (0.5–1.6)	**<0.001**
	Myo (ng/mL)	34.9 (26.0–57.4)	53.7 (33.6–93.4)	**<0.001**	29.8 (23.4–41.2)	38.1 (27.4–60.2)	**<0.001**
	NT-proBNP (pg/mL)	80.0 (30.0–198.5)	352.5 (109.5–1291.5)	**<0.001**	63.0 (26.0–156.5)	267.5 (82.3–1198.8)	**<0.001**
Alive		*n* = 854	*n* = 109		*n* = 854	*n* = 109	
	Hs-TnI (pg/mL)	2.5 (1.9–6.5)	7.6 (3.1–19.7)	**<0.001**	1.9 (1.9–4.3)	5.3 (2.5–13.0)	**<0.001**
	CK-MB (ng/mL)	0.7 (0.5–1.1)	1.1 (0.7–1.9)	**<0.001**	0.6 (0.4–0.8)	0.9 (0.5–1.3)	**<0.001**
	Myo (ng/mL)	34.3 (25.7–53.2)	47.5 (32.3–76.2)	**<0.001**	29.1 (23.1–38.5)	33.8 (25.0–50.2)	**0.002**
	NT-proBNP (pg/mL)	72.0 (29.0–173.5)	257.0 (94.0–1101.0)	**<0.001**	57.0 (25.0–134.3)	169.0 (65.5–533.5)	**<0.001**
Died		n = 43	n = 17		n = 43	n = 17	
	Hs-TnI (pg/mL)	35.3 (5.5–296.4)	16.7 (9.4–53.2)	0.337	156.1 (32.6–523.5)	107.4 (29.2–748.7)	0.658
	CK-MB (ng/mL)	2.9 (1.2–4.6)	1.0 (0.5–2.4)	**0.007**	4.5 (2.1–10.2)	6.4 (1.7–11.6)	0.688
	Myo (ng/mL)	174.1 (108.4–368.6)	101.9 (76.2–197.6)	0.070	869.8 (368.6–1200.0)	587.3 (211.5–1200.0)	0.416
	NT-proBNP (pg/mL)	1032.0 (359.0–3122.0)	991.0 (676.0–2970.5)	0.583	4985.0 (1824.0–10957.0)	2596.0 (1207.5–12869.5)	0.528

*p values were calculated between the cardiac and non-cardiac groups by Mann-Whitney U test*.

*Hs-TnI, High sensitivity troponin-I; CK-MB, creatine kinase-MB; Myo, myoglobin; NT-proBNP, N terminal pro B type natriuretic peptide. The bold p values mean that they are of significance in statistics*.

As shown, the levels of biomarkers in cardiac survivors were significantly higher than those in non-cardiac survivors at both the early and late stages of illness. However, the levels of biomarkers in cardiac non-survivors were comparable to those in non-survivors without concomitant cardiac disease. There is a significant downward trend of CK-MB level in cardiac non-survivors compared to non-survivors without cardiac disease in the early stage (*p* = 0.007). No significant differences were found in the levels of Hs-TnI, Myo, and NT-proBNP between the two groups at both the early and late stages of disease (Figures 1, 2 and Table 2).

### Cardiac Biomarker Levels and Clinical Characteristics of Survivors and Non-survivors Among Cardiac and Non-cardiac Patients

The levels of cardiac biomarkers in the overall study population, cardiac, non-cardiac, and patients stratified by mortality were summarized in Supplementary Table 3. Concentrations of all four biomarkers in non-survivors were significantly higher than that in survivors in the overall study population, cardiac patients, and non-cardiac patients, except that the level of CK-MB in cardiac non-survivors was comparable to the cardiac survivors at the early stage of disease. The clinical characteristics and laboratory results of survivors and non-survivors among the overall study population, cardiac and non-cardiac patients were listed in Supplementary Tables 4–6, respectively.

### Comparison of the Cardiac Biomarker Levels Between the Early and the Late Stages Among Survivors and Non-survivors, Stratified by Concomitant Cardiac Disease

The levels of cardiac biomarkers at the early and the late stages in the overall study population, survivors, non-survivors, and patients stratified by concomitant cardiac disease were summarized in Supplementary Table 7 and Supplementary Figure 1. As shown, the cardiac biomarker levels at the late stage were significantly decreased compared to those at the early stage among patients who were alive. Whereas, the late-stage biomarker levels were significantly increased in patients who ultimately died.

### Cardiac Biomarker Levels at the Early and the Late Stages in Patients Stratified Based on Clinical Classifications

**Figure 3:**
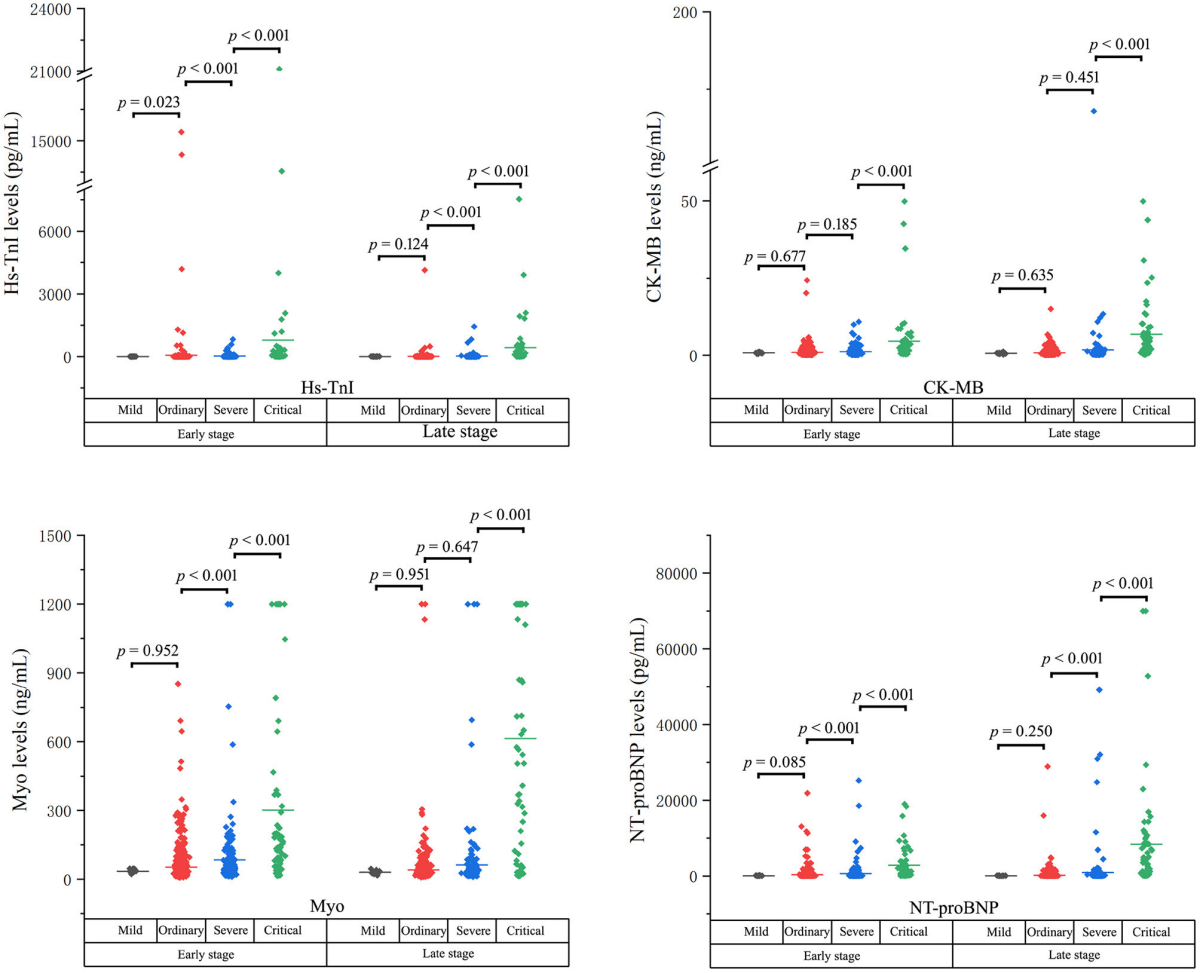
The cardiac biomarker levels at both the early and the late stages in patients stratified based on clinical classifications.

The cardiac biomarker levels at the early and the late stages in patients stratified based on clinical classifications were illustrated in Table 3 and Figure 3. The levels of cardiac biomarkers in ordinary cases were comparable to that in mild cases at both the early and late stages, except that the early-stage level of Hs-TnI was higher in ordinary cases. The levels of Hs-TnI and NT-proBNP at both the early and late stages and the level of Myo at the early stage were significantly higher in severe patients than that in ordinary cases. When compared with severe cases, critical patients had significantly elevated levels of cardiac biomarkers at both the early and late stages. Overall, these results suggest that the increases in cardiac biomarkers were closely related to the severity of the disease.

**Table 3:** Cardiac biomarker levels at the early and late stages in patients stratified by clinical classifications.

**Classifications**	**Biomarkers**	**Early stage**		**Late stage**	
		**Biomarker levels**	***p***	**Biomarker levels**	***p***
Mild (*n* = 14)	Hs-TnI (pg/mL)	1.9 (1.9–2.4)	**-**	1.9 (1.9–2.3)	-
	CK-MB (ng/mL)	0.8 (0.6–0.9)	**-**	0.6 (0.5–0.9)	-
	Myo (ng/mL)	33.9 (30.8–41.8)	**-**	30.2 (26.4–36.4)	-
	NT-proBNP (pg/mL)	47.0 (13.0–134.5)	**-**	58.0 (10.0–128.3)	-
Ordinary (*n* = 742)	Hs-TnI (pg/mL)	2.4 (1.9–6.6)	0.023^^*^^	1.9 (1.9–4.7)	0.124^^*^^
	CK-MB (ng/mL)	0.7 (0.5–1.1)	0.677^^*^^	0.6 (0.4–0.9)	0.635^^*^^
	Myo (ng/mL)	33.9 (25.7–52.6)	0.952^^*^^	29.7 (23.4–39.7)	0.951^^*^^
	NT-proBNP (pg/mL)	72.5 (29.0–181.8)	0.085^^*^^	59.0 (25.0–153.3)	0.250^^*^^
Severe (*n* = 205)	Hs-TnI (pg/mL)	4.5 (2.5–12.1)	**<0.001**^**^#^**^	2.8 (1.9–6.8)	**<0.001**^**^#^**^
	CK-MB (ng/mL)	0.8 (0.5–1.3)	0.185^**^#^**^	0.6 (0.4–1.0)	0.451^**^#^**^
	Myo (ng/mL)	41.5 (28.4–78.2)	**<0.001**^**^#^**^	29.5 (22.8–41.6)	0.647^**^#^**^
	NT-proBNP (pg/mL)	122.0 (55.0–401.0)	**<0.001**^**^#^**^	88.0 (39.0–199.5)	**<0.001**^**^#^**^
Critical (*n* = 62)	Hs-TnI (pg/mL)	23.9 (7.7–215.4)	**<0.001**^**^†^**^	74.0 (20.0–434.8)	**<0.001**^**^†^**^
	CK-MB (ng/mL)	2.3 (0.9–3.8)	**<0.001**^**^†^**^	3.1 (1.0–7.3)	**<0.001**^**^†^**^
	Myo (ng/mL)	138.8 (79.4–331.1)	**<0.001**^**^†^**^	554.6 (77.1–1200.0)	**<0.001**^**^†^**^
	NT-proBNP (pg/mL)	1023.0 (358.0–3293.5)	**<0.001**^**^†^**^	4148.5 (706.5–9920.3)	**<0.001**^**^†^**^

*p values were calculated between the groups by Mann-Whitney U test*.

* *Comparison with the mild group*.

# *Comparison with the ordinary group*.

† *Comparison with the severe group*.

*Hs-TnI, High sensitivity troponin-I; CK-MB, creatine kinase-MB; Myo, myoglobin; NT-proBNP, N terminal pro B type natriuretic peptide. The bold p values mean that they are of significance in statistics*.

### Cumulative Survival Curves of in-hospital Death in COVID-19 Patients With and Without Cardiac Disease

**Figure 4:**
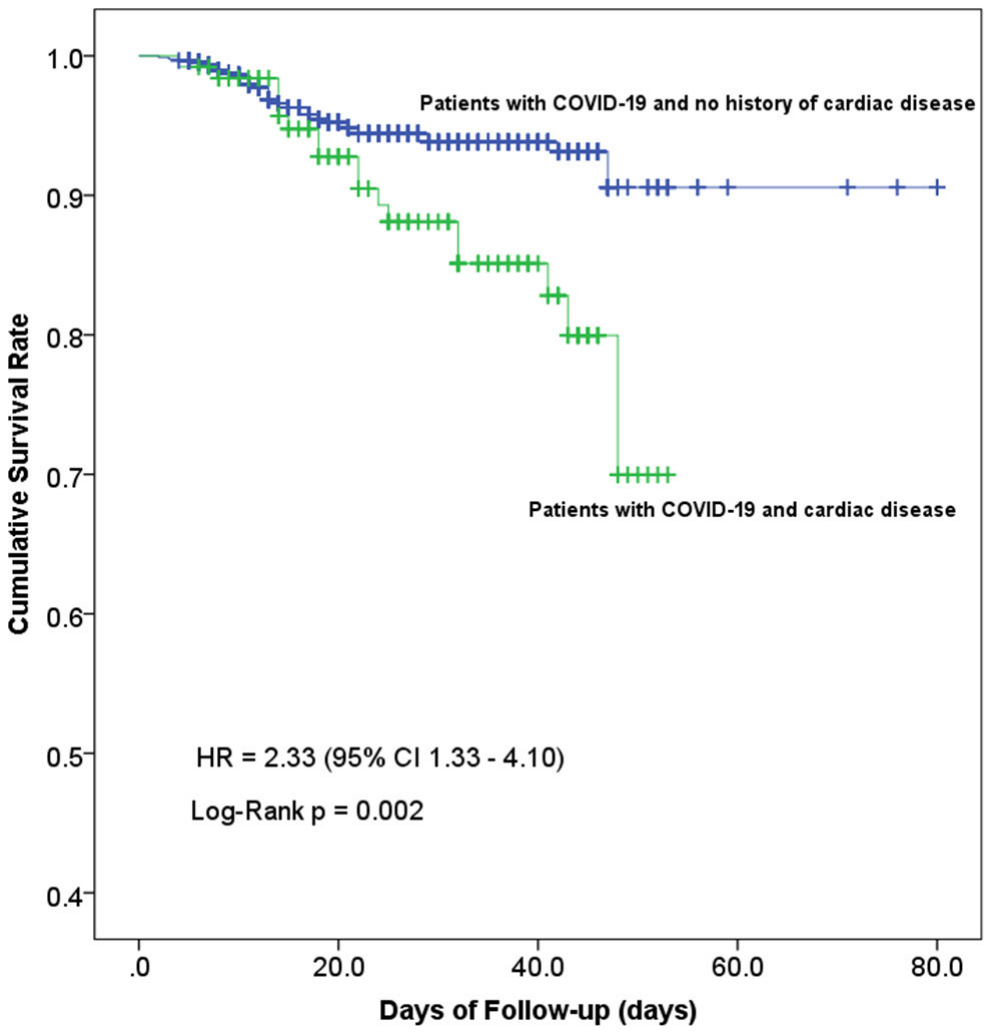
Kaplan-Meier curves showing the cumulative survival rate of COVID-19 patients with and without concomitant cardiac disease. Line in blue, patients with cardiac disease, *n* = 126; line in green, patients without cardiac disease, *n* = 897; log-rank test for trend, *p* = 0.002.

Cumulative survival rate curves of in-hospital death were calculated using the Kaplan-Meier product-limit estimation method with the log-rank test. As shown in Figure 4, the risk of in-hospital mortality was significantly higher in cardiac patients compared with non-cardiac patients (log-rank *p* = 0.002; HR 2.33; 95% confidence interval 1.33–4.10).

### Receiver Operator Characteristic (ROC) Curves Demonstrating the Ability of Cardiac Biomarkers for Predicting in-hospital Mortality

**Figure 5:**
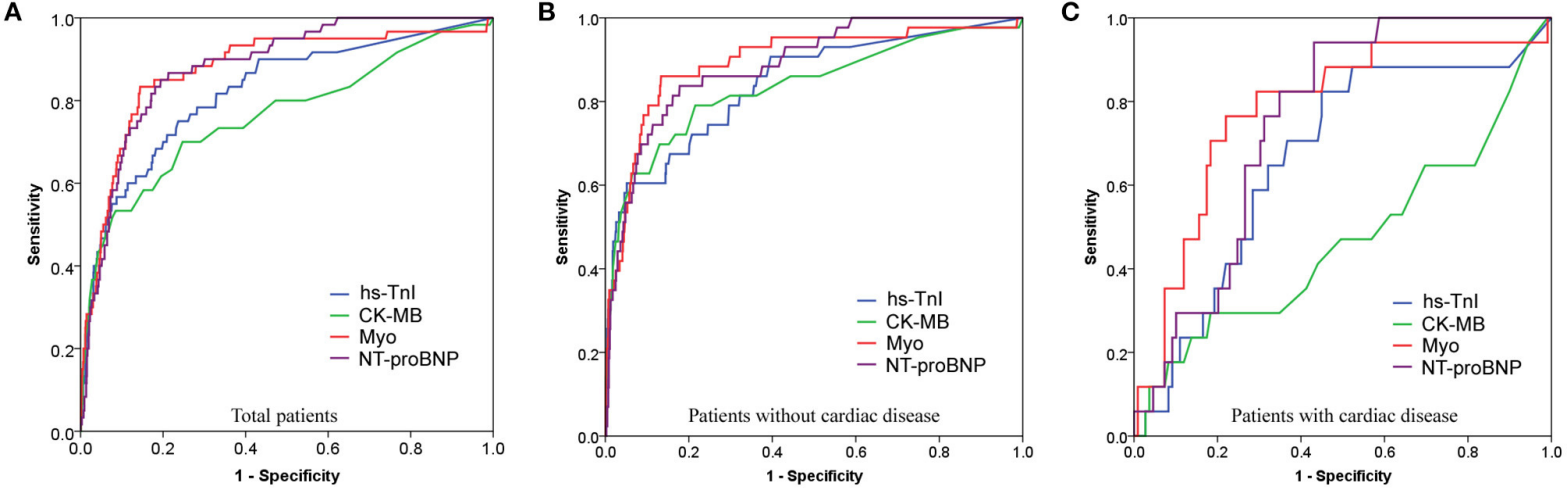
Receiver operating characteristic (ROC) curves showing the prognostic value of cardiac biomarkers for predicting in-hospital death in overall study population **(A)**, non-cardiac **(B)**, and cardiac **(C)** patients.

As illustrated above, the cardiac biomarker levels were significantly different between cardiac and non-cardiac patients. It is reasonable that the prognostic ability of them in between cardiac and non-cardiac patients could be different. The ROC performance of each biomarker for predicting in-hospital mortality in the overall study population, cardiac, and non-cardiac patients was shown in Figure 5 and Table 4.

**Table 4:** Overall performance of cardiac biomarkers for predicting in-hospital mortality in overall study population, cardiac, and non-cardiac patients according to ROC curves.

	**Biomarkers**	**AUC (95% CI)**	***p* value**	**Cut-off**	**Sensitivity, %**	**Specificity, %**
All included patients (*n* = 1,023)	Hs-TnI	0.822 (0.761–0.882)	<0.001	7.85 pg/mL	75.0	76.3
	CK-MB	0.763 (0.689–0.838)	<0.001	1.15 ng/mL	70.0	75.3
	Myo	0.875 (0.822–0.928)	<0.001	80.55 ng/mL	83.3	85.6
	NT-proBNP	0.881 (0.841–0.920)	<0.001	301.5 pg/mL	85.0	80.6
Non-cardiac patients (*n* = 897)	Hs-TnI	0.842 (0.774–0.911)	<0.001	22.95 pg/mL	60.5	94.8
	CK-MB	0.837 (0.761–0.913)	<0.001	1.15 ng/mL	79.1	78.5
	Myo	0.895 (0.837–0.953)	<0.001	80.55 ng/mL	86.0	86.7
	NT-proBNP	0.888 (0.839–0.936)	<0.001	266.0 pg/mL	83.7	82.2
Cardiac patients (*n* = 126)	Hs-TnI	0.664 (0.528–0.801)	0.030	8.65 pg/mL	82.4	55.1
	CK-MB	0.532 (0.366–0.697)	0.674	ns	-	-
	Myo	0.778 (0.655–0.902)	<0.001	82.10 ng/mL	76.5	78.0
	NT-proBNP	0.753 (0.659–0.847)	0.001	352.5 pg/mL	94.1	56.9

*ns indicates non-significant*.

*AUC, area under the ROC curves; Hs-TnI, High sensitivity troponin-I; CK-MB, creatine kinase-MB; Myo, myoglobin; NT-proBNP, N terminal pro B type natriuretic peptide*.

The area under the ROC curve (AUC) of Hs-TnI, CK-MB, Myo, and NT-proBNP for the in-hospital mortality in the overall study population was 0.822 (95%CI, 0.761–0.882), 0.763 (95%CI, 0.689–0.838), 0.875 (95%CI, 0.822–0.928), and 0.881 (95%CI, 0.841–0.920), respectively, all with significant sensitivity and specificity (all *p* < 0.001). The best cut-off point of Hs-TnI, CK-MB, Myo, and NT-proBNP was 7.85 pg/mL, 1.15 ng/mL, 80.55 ng/Ml, and 301.5 pg/mL, respectively.

For non-cardiac patients, the AUC of Hs-TnI, CK-MB, Myo, and NT-proBNP was 0.842 (95%CI, 0.774–0.911), 0.837 (95%CI, 0.761–0.913), 0.895 (95%CI, 0.837–0.953), and 0.888 (95%CI, 0.839–0.936), respectively, all with significant sensitivity and specificity (all *p* < 0.001). The best cut-off point of Hs-TnI, CK-MB, Myo, and NT-proBNP was 22.95 pg/mL, 1.15 ng/mL, 80.55 ng/mL, and 266.0 pg/mL, respectively.

As for cardiac patients, the AUC of Hs-TnI, CK-MB, Myo, and NT-proBNP was 0.664 (95%CI, 0.528–0.801), 0.532 (95%CI, 0.366–0.697), 0.778 (95%CI, 0.655–0.902) and 0.753 (95%CI, 0.659–0.847), respectively. There were significant differences for Hs-TnI, Myo, and NT-proBNP. Whereas, no significant difference was found in CK-MB (*p* = 0.674). The cut-off point of Hs-TnI, Myo, and NT-proBNP was 8.65 pg/mL, 82.10 ng/mL, and 352.5 pg/mL, respectively.

These results suggest that Myo, an early release biomarker of cardiac injury, had the highest overall performance to predict the in-hospital mortality of COVID-19, followed by NT-proBNP, and Hs-TnI. CK-MB showed the lowest prognostic performance. More importantly, cardiac biomarkers are prognostic for in-hospital mortality in non-cardiac than in cardiac patients.

### Cumulative Survival Curves of Patients With Cardiac Biomarker Level Under and Above Cut-Offs

**Figure 6:**
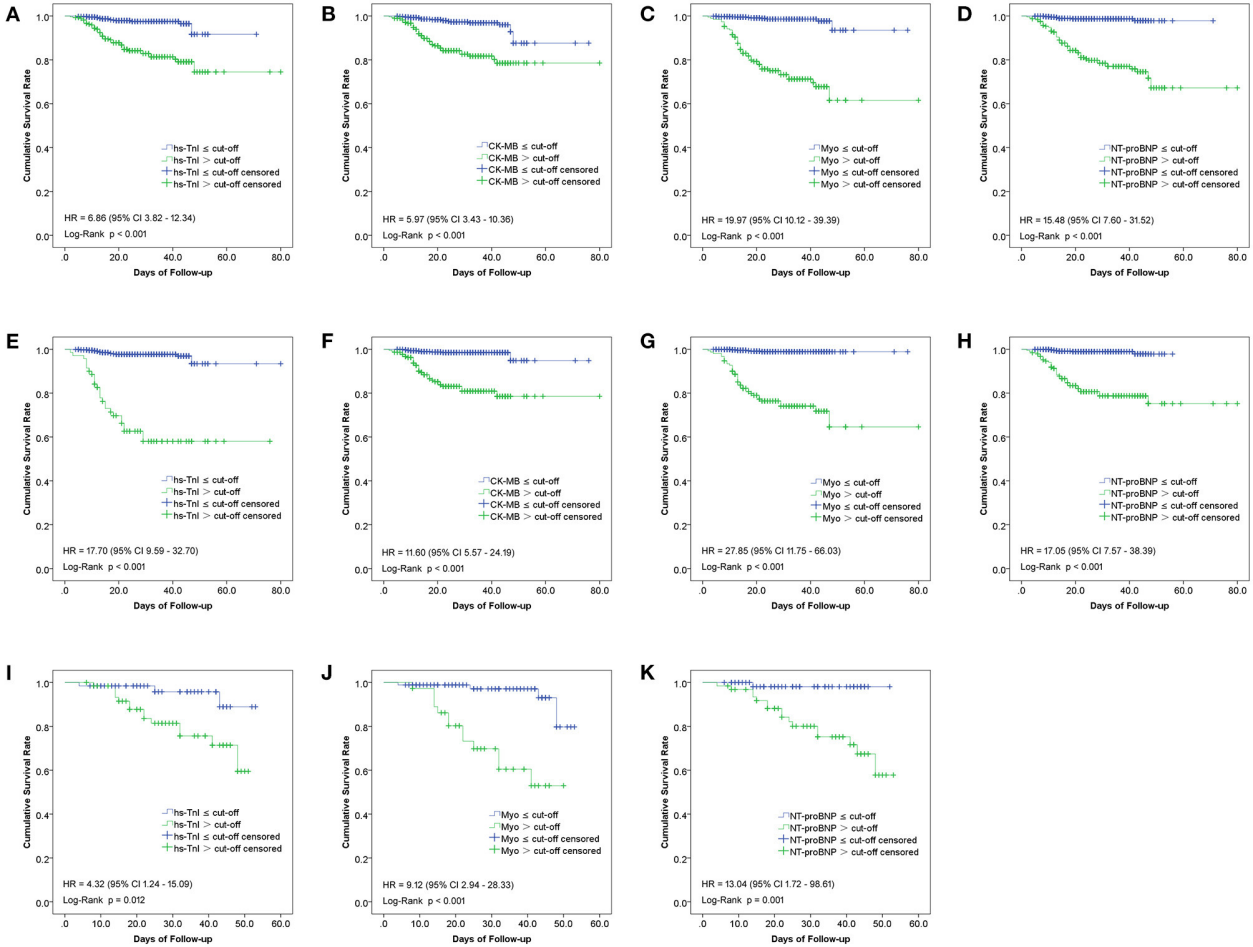
Kaplan-Meier curves between groups categorized by the cut-off value of each biomarker in the overall study population **(A–D)**, non-cardiac **(E–H)**, and cardiac **(I–K)** patients.

The Kaplan-Meier curves between groups categorized by the cut-off value of each biomarker in the overall study population, cardiac, and non-cardiac patients were shown in Figure 6. As illustrated, patients with Hs-TnI, CK-MB, Myo, and NT-proBNP levels above the cut-offs had significantly decreased survival rates (Figure 6).

The risks for in-hospital mortality of these biomarkers were estimated using Cox proportional hazards regression analysis. For this purpose, the value of each biomarker was transformed into a categorical variable according to the ROC cut-off point. The HRs for associations of in-hospital mortality of overall study population patients with increased Hs-TnI, CK-MB, Myo, and NT-proBNP, was 6.86 (95%CI, 3.82–12.34), 5.97 (95%CI, 3.43–10.36), 19.97 (95%CI, 10.12–39.39), and 15.48 (95%CI, 7.60–31.52), respectively. For non-cardiac patients, the HRs of Hs-TnI, CK-MB, Myo, and NT-proBNP were 17.70 (95%CI, 9.59–32.70), 11.60 (95%CI, 5.57–24.29), 27.85 (95%CI, 11.75–66.03) and 17.05 (95%CI, 7.57–38.39), respectively. As for cardiac patients, the HRs of Hs-TnI, Myo, and NT-proBNP were 4.32 (95%CI, 1.24–15.09), 9.12 (95%CI, 2.94–28.33), and 13.04 (95%CI, 1.72–98.61), respectively. Elevation of Myo exhibited the highest HR, followed by NT-ProBNP and Hs-TnI. CK-MB had the lowest HR with insufficient predictive capacity for cardiac patients (Figure 6).

### Cox Proportional Hazards Regression Analyses

Univariate Cox regression analyses were conducted to evaluate the association between variables and the risk of in-hospital mortality in the overall study population (Supplementary Table 8). Age, SpO_2_, cardiac biomarkers (Hs-TnI, CK-MB, Myo, and NT-proBNP), inflammatory indicators (WBC, NEU, LYM, Hs-CRP, IL2R, IL6, IL8, and TNFα), and coagulation index (PLT, D-dimer, and INR) were of strong prognostic power (reflected in high Wald values and significant hazard ratios).

Given the significant associations of the cardiac biomarkers with in-hospitalization mortality in univariate Cox analyses, we developed multivariate models controlled for potential confounders such as age, sex, comorbidities, inflammatory and coagulation factors (NEU, Hs-CRP, IL-2R, PLT, and D-dimer). These confounders were included according to the results of univariate Cox regression analyses, in which they had relatively higher Wald values.

We found that after these potential confounders were controlled for, Myo and NT-proBNP were still significantly associated with in-hospital mortality in the overall study population and non-cardiac patients. Hs-TnI and CK-MB in the crude mortality rate of the overall study population and non-cardiac patients were statistically significant, but these associations disappeared after the confounders were taken into consideration. For the cardiac patients, all the biomarkers were not associated with in-hospital mortality after the confounders were adjusted for (Table 5).

**Table 5:** Multivariate Cox proportional-hazards regression analyses of cardiac biomarkers on risk of in-hospital death in all study population, cardiac, and non-cardiac patients.

	**Biomarkers**	**Unadjusted**		**Model 1**		**Model 2**		**Model 3**	
		**HR (95% CI)**	***p***	**HR (95% CI)**	***p***	**HR (95% CI)**	***p***	**HR (95% CI)**	***p***
All included patients (*n* = 1,023)	Hs-TnI, per 10 pg/mL	1.002 (1.001–1.003)	**<0.001**	1.002 (1.001–1.002)	**<0.001**	1.002 (1.001–1.002)	**<0.001**	1.001 (1.000–1.002)	0.184
	CK-MB, per 1 ng/mL	1.078 (1.053–1.103)	**<0.001**	1.047 (1.021–1.073)	**<0.001**	1.052 (1.022–1.083)	**0.001**	0.996 (0.964–1.030)	0.825
	Myo, per 10 ng/mL	1.029 (1.023–1.035)	**<0.001**	1.024 (1.017–1.030)	**<0.001**	1.026 (1.019–1.032)	**<0.001**	1.010 (1.002–1.017)	**0.012**
	NT-proBNP, per 100 pg/mL	1.015 (1.010–1.019)	**<0.001**	1.012 (1.008–1.017)	**<0.001**	1.013 (1.008–1.018)	**<0.001**	1.006 (1.000–1.013)	**0.049**
Non-cardiac patients (*n* = 897)	Hs-TnI, per 10 pg/mL	1.002 (1.001–1.003)	**<0.001**	1.002 (1.001–1.002)	**<0.001**	1.002 (1.001–1.002)	**<0.001**	1.001 (1.000–1.001)	0.172
	CK-MB, per 1 ng/mL	1.117 (1.088–1.146)	**<0.001**	1.097 (1.067–1.129)	**<0.001**	1.093 (1.062–1.126)	**<0.001**	1.000 (0.959–1.043)	0.990
	Myo, per 10 ng/mL	1.031 (1.024–1.037)	**<0.001**	1.026 (1.019–1.032)	**<0.001**	1.029 (1.021–1.036)	**<0.001**	1.009 (1.000–1.018)	**0.038**
	NT-proBNP, per 100 pg/mL	1.018 (1.012–1.023)	**<0.001**	1.018 (1.013–1.024)	**<0.001**	1.018 (1.011–1.024)	**<0.001**	1.010 (1.001–1.020)	**0.034**
Cardiac patients (*n* = 126)	Hs-TnI, per 10 pg/mL	0.999 (0.990–1.008)	0.781	0.998 (0.986–1.010)	0.709	0.998 (0.986–1.010)	0.722	0.992 (0.977–1.007)	0.308
	CK-MB, per 1 ng/mL	0.996 (0.900–1.101)	0.934	0.989 (0.897–1.090)	0.822	0.989 (0.892–1.097)	0.830	0.947 (0.854–1.050)	0.300
	Myo, per 10 ng/mL	1.019 (1.001–1.036)	**0.037**	1.014 (0.995–1.032)	0.146	1.018 (0.996–1.041)	0.116	1.003 (0.978–1.028)	0.834
	NT-proBNP, per 100 pg/mL	1.009 (1.001–1.018)	**0.025**	1.008 (1.000–1.016)	0.056	1.007 (0.999–1.016)	0.101	1.002 (0.991–1.013)	0.719

*Model 1: Adjusted for age and sex. Model 2: Model 1 plus comorbidities (cardiac disease, hypertension, diabetes, chronic liver disease, stroke, chronic kidney disease, chronic obstructive pulmonary disease, and cancer). Model 3: Model 2 plus inflammatory and coagulation factors (platelet, D-dimer, neutrophil, high sensitivity C-reactive protein, and interleukin 2 receptor). HR, hazards ratio; CI, confidence interval; Hs-TnI, High sensitivity troponin-I; CK-MB, creatine kinase-MB; Myo, myoglobin; NT-proBNP, N terminal pro B type natriuretic peptide. The bold p values mean that they are of significance in statistics*.

## Discussion

This study aimed to investigate the differences regarding the release pattern and prognostic values of cardiac biomarkers in between cardiac and non-cardiac patients with COVID-19. The significant findings are as follows: (1) Among the overall study population and survived patients, the cardiac biomarker levels at both the early and late stages in cardiac patients were significantly higher than those in non-cardiac ones. However, their concentrations in cardiac patients were comparable to non-cardiac ones among non-survivors. (2) The levels of cardiac biomarkers at the late stage were significantly decreased compared with those at the early stage in patients who were alive. Whereas, the late-stage biomarker levels were significantly increased among patients who ultimately died. (3) Subgroup analysis illustrated that increases in cardiac biomarkers were closely related to the severity of the disease, and were prognostic for high risks of in-hospital mortality in non-cardiac, rather than in cardiac patients. Myo had the highest overall prognostic performance, followed by NT-proBNP and Hs-TnI. CK-MB manifested the lowest performance. (4) Myo and NT-proBNP, rather than Hs-TnI and CK-MB, were independently associated with in-hospital mortality in the overall population and non-cardiac patients. However, these associations were not significant among cardiac patients.

Mounting evidence substantiated myocardial injury, manifested by elevation of cardiac biomarkers, is a common condition among hospitalized patients with COVID-19 (2, 5, 13–16). Increased levels of cardiac biomarkers are associated with more severe clinical course and adverse clinical outcomes (10, 17, 18). The exact mechanism of cardiac biomarker elevation after COVID-19 infection is not fully understood. But myocarditis, stress cardiomyopathy, acute heart failure, and direct injury from SARS-CoV-2 were supposed to be essential etiologies (6). The receptor for SARS-CoV-2, ACE2, is expressed on vascular endothelial cells and myocytes, so there is at least theoretical potential possibility of direct cardiovascular involvement by the virus (10). Other proposed mechanisms include a cytokine storm triggered by an imbalanced response by type 1 and type 2 T helper cells, increased prothrombotic and procoagulant responses following SARS-CoV-2 infection, respiratory dysfunction with hypoxemia, and hemodynamic instability caused by COVID-19, resulting in damage to myocardial cells (6, 15).

Our findings are consistent with previous reports that COVID-19 patients with a history of cardiac disease had higher levels of cardiac biomarkers and poorer outcomes than patients without a history of cardiac disease (9). The mechanism of poorer prognosis in patients with concomitant cardiac disease may be complicated. In this cohort, compared with non-cardiac COVID-19 patients, cardiac patients were older (72 [64-80] vs. 61 [50-69] years; *p* < 0.001) and of higher prevalence of comorbidities, which were consistently shown to be the significant risk factors for myocardial injury and poor outcomes (7, 19). It is worth stressing that older age is an important factor contributing to the adverse outcomes of COVID-19 patients. COVID-19 patients with older age were proved to have significantly higher body temperature, co-existing of basic diseases, and rate of severe and critical type during hospitalization (20). We also demonstrated that cardiac patients presented a higher number of white blood cells, more severe lymphopenia, elevated levels of inflammatory factors, and increased coagulation index, which indicated an exaggerated inflammatory activation, hypercoagulable state, and hemostatic abnormality in these patients. This may partially explain the susceptibility of cardiac patients to COVID-19 and their poorer prognosis, as exaggerated immune response with microthrombi formation was proved to be a vital mechanism of the high mortality of COVID-19 (21–24).

The novel finding of our study is that the levels of cardiac biomarkers in cardiac non-survivors were comparable to those dying without cardiac disease at both the early and late stages of illness. It's notable that, among non-survivors, cardiac patients had relatively higher levels of SpO_2_ and platelet counts, and lower levels of Hs-CRP and IL-8 at admission than non-cardiac ones, which were of opposite trends in that among the survivors. The origin of this phenomenon was unknown, but this finding indicates, at least, that cardiac patients who ultimately died did not develop more severe myocardial injury and/or inflammatory activation at admission than non-cardiac ones.

Our study demonstrated for the first time that cardiac biomarkers had a better performance for predicting in-hospital mortality in non-cardiac patients than in cardiac patients. Myo and NT-proBNP had relatively more robust prognostic power than Hs-TnI and CK-MB, which was not noticed in previous studies. Myo and NT-proBNP had a larger area under the curve in the ROC analysis. More importantly, Myo and NT-proBNP, rather than Hs-TnI and CK-MB, were the independent factors associated with the risk of in-hospital death after adjusting for other confounders in the multivariate Cox regression analyses. These results differ from an earlier report that Hs-TnI, Myo, NT-proBNP, and CK-MB had comparable prognostic power and all of them were significantly associated with increased mortality risk of COVID-19 patients (11). In the study conducted by Qin et al. the authors did not conduct multivariate analysis to further control for potential confounders, and their follow-up time was 28 days, which was different from our study (11). Overall, our results suggest that increased levels of Myo and NT-proBNP on admission could be useful markers for early identifying high-risk patients. However, special attention must be paid when implementing their prognostic function for cardiac patients.

Despite the value of these findings, our study has some limitations. First, this study is based on the extraction of data from medical records. Despite our efforts to include all qualified patients, some patients were excluded due to the absence of relevant clinical data. Second, because the time from diagnosis to admission varies among enrolled patients, the levels of clinical variables on admission might be influenced. Third, the data of the biomarkers was only early single time point measurements. The dynamic variation of these biomarkers during hospitalization was not studied in our research, which needs to be evaluated in further studies. Finally, this is a single-center study. For total patients with COVID-19 worldwide, the current sample size is still small. Further studies involving additional populations and multiple centers are warranted. Nevertheless, by understanding the release and prognostic values of cardiac biomarkers, it might help clinicians to early identify high-risk patients and to improve outcomes by making corresponding decisions.

## Data Availability Statement

The original contributions presented in the study are included in the article/Supplementary Materials, further inquiries can be directed to the corresponding author/s.

## Ethics Statement

The studies involving human participants were reviewed and approved by the ethics committee of Tongji hospital, Huazhong University of Science and Technology. Written informed consent for participation was not required for this study in accordance with the national legislation and the institutional requirements.

## Author Contributions

J-SY and HL designed the study, analyzed data, and wrote the manuscript. N-NP and R-DC collected and reviewed clinical and laboratory data. HL and L-CZ performed the statistical analysis. HL and H-KY edited manuscript. J-SY, N-NP, and HL reviewed, interpreted, and checked clinical data. All authors contributed to the article and approved the submitted version.

## Conflict of Interest

The authors declare that the research was conducted in the absence of any commercial or financial relationships that could be construed as a potential conflict of interest.

## Abbreviations

COVID-19: coronavirus disease 2019
SARS-CoV-2: severe acute respiratory syndrome coronavirus 2
ACE2: angiotensin-converting enzyme 2
IQR: interquartile range
HP: hypertension
DM: diabetes
COPD: chronic obstructive pulmonary disease
SBP: Systolic blood pressure
DBP: Diastolic blood pressure
SpO_2_: percutaneous oxygen saturation
Hs-TnI: High sensitivity troponin-I
CK-MB: creatine kinase-MB
Myo: myoglobin
NT-proBNP: N terminal pro B type natriuretic peptide
WBC: white blood cell
NEU: neutrophil
NEU%: neutrophil percentage
LYM: lymphocytes
LYM%: lymphocyte percentage
Hs-CRP: high sensitivity C-reactive protein
IL2R: interleukin 2 receptor
IL6: interleukin 6
IL8: interleukin 8
TNFα: tumor necrosis factor α
PLT: platelet
FIB: fibrinogen
INR: international normalized ratio
ALT: alanine aminotransferase
AST: aspartate transaminase
ALB: albumin
GLOB: globulin
Cr: creatinine
EGFR: estimated glomerular filtration rate
GLU: glucose
TBIL: total bilirubin.
