# Supplementary material for: Cardiac Biomarker Levels and Their Prognostic Values in COVID-19 Patients With or Without Concomitant Cardiac Disease
Source: Front Cardiovasc Med. 2021 Jan 20;7:599096. doi: 10.3389/fcvm.2020.599096 (PMC7856675; doi:10.3389/fcvm.2020.599096)
Supplement: Supplementary file 1 [file Table_1.DOCX]

Table S1. Clinical characteristics and on-admission laboratory findings of survived patients stratified based on concomitant cardiac disease.

| **Characteristics** | **Total survivors**  **(n = 963)** | **Survivors without cardiac disease**  **(n = 854)** | **Survivors with cardiac disease**  **(n = 109)** | ***p* value** |
| --- | --- | --- | --- | --- |
| **Age (yrs), median (IQR)** | 62 (50 - 69) | 60 (49 - 68) | 71 (64 - 80) | **< 0.001** |
| **Male/Female, n** | 448/515 | 397/457 | 51/58 | 0.953 |
| **Comorbidities, n (%)** | | | | |
| History of HP-n (%) | 341 (35.4) | 261 (30.6) | 80 (73.4) | **< 0.001** |
| History of DM -n (%) | 156 (16.2) | 124 (14.5) | 32 (29.4) | **< 0.001** |
| Chronic liver disease-n (%) | 14 (1.5) | 14 (1.6) | 0 (0) | 0.178 |
| Stroke history-n (%) | 41 (4.3) | 32 (3.7) | 9 (8.3) | **0.028** |
| Chronic kidney disease-n (%) | 20 (2.1) | 15 (1.8) | 5 (4.6) | 0.051 |
| History of COPD-n (%) | 10 (1.0) | 7 (0.8) | 3 (2.8) | 0.061 |
| Cancer-n (%) | 35 (3.6) | 27 (3.2) | 8 (7.3) | **0.028** |
| **Clinical classifications, n (%)** | | | | |
| Mild cases-n (%) | 14 (1.5) | 14 (1.6) | 0 (0) | 0.178 |
| Ordinary cases-n (%) | 735 (76.3) | 661 (77.4) | 74 (67.9) | **0.028** |
| Severe cases-n (%) | 198 (20.6) | 168 (19.7) | 30 (27.5) | 0.056 |
| Critical cases-n (%) | 16 (1.7) | 11 (1.3) | 5 (4.6) | **0.011** |
| **Physical examination on admission, median (IQR)** | | | | |
| Temperature (°C) | 36.5 (36.2 - 36.9) | 36.5 (36.2 - 36.8) | 36.5 (36.3 - 37.0) | 0.797 |
| Pulse (/min) | 89 (80 - 100) | 90 (80 - 100) | 87 (76 - 100) | 0.070 |
| Respire (/min) | 20 (19 - 22) | 20 (19 - 22) | 20 (19 - 24) | 0.192 |
| SBP (mmHg) | 133 (120 - 145) | 132 (120 - 145) | 138 (122 - 150) | **0.012** |
| DBP (mmHg) | 81 (73 - 90) | 81 (73 - 90) | 78 (72 - 88) | 0.246 |
| SpO_2_ (%) | 97 (95 - 98) | 97 (95 - 98) | 97 (95 - 98) | 0.340 |
| **Laboratory tests on admission, median (IQR)** | | | | |
| Hs-TnI (pg/mL) | 2.8 (1.9 - 7.6) | 2.5 (1.9 - 6.5) | 7.6 (3.1 - 19.7) | **< 0.001** |
| CK-MB (ng/mL) | 0.7 (0.5 - 1.1) | 0.7 (0.5 - 1.1) | 1.1 (0.7 - 1.9) | **< 0.001** |
| Myo (ng/mL) | 35.0 (26.5 - 55.6) | 34.3 (25.7 - 53.2) | 47.5 (32.3 - 76.2) | **< 0.001** |
| NT-proBNP (pg/mL) | 83.0 (32.0 - 210.0) | 72.0 (29.0 - 173.5) | 257.0 (94.0 - 1101.0) | **< 0.001** |
| WBC (10^9/L) | 5.90 (4.73 - 7.38) | 5.86 (4.67 - 7.34) | 6.09 (5.08 - 7.94) | **0.024** |
| NEU (10^9/L) | 3.69 (2.71 - 5.08) | 3.61 (2.67 - 4.96) | 4.25 (3.11 - 5.77) | **< 0.001** |
| NEU% (%) | 63.3 (55.5 - 72.1) | 62.7 (55.1 - 71.6) | 69.3 (60.0 - 78.8) | **< 0.001** |
| LYM (10^9/L) | 1.36 (0.98 - 1.80) | 1.38 (1.01 - 1.83) | 1.13 (0.77 - 1.59) | **< 0.001** |
| LYM% (%) | 25.0 (17.0 - 31.8) | 25.8 (17.8 - 32.2) | 18.9 (11.7 - 26.5) | **< 0.001** |
| Hs-CRP (mg/L) | 4.5 (1.2 - 31.6) | 4.0 (1.0 - 27.4) | 13.5 (2.7 - 44.7) | **< 0.001** |
| IL2R (U/mL) | 465.0 (309.0 - 716.0) | 448.5 (299.5 - 693.3) | 651.0 (436.5 - 995.0) | **< 0.001** |
| IL6 (pg/mL) | 3.63 (1.70 - 10.09) | 3.35 (1.59 - 8.72) | 5.61 (3.13 - 20.43) | **< 0.001** |
| IL8 (pg/mL) | 9.9 (6.5 - 16.9) | 9.4 (6.4 - 16.1) | 13.1 (8.4 - 22.5) | **< 0.001** |
| TNFα (pg/mL) | 8.1 (6.2 - 10.4) | 8.0 (6.1 - 10.2) | 9.6 (7.3 - 12.2) | **< 0.001** |
| PLT (10^9/L) | 234 (188 - 302) | 236 (191 - 303) | 220 (170 - 300) | 0.055 |
| D-dimer (μg/mL FEU) | 0.51 (0.23 - 1.20) | 0.49 (0.22 - 1.11) | 0.78 (0.38 - 2.30) | **< 0.001** |
| FIB (g/L) | 4.03 (3.19 - 5.35) | 4.01 (3.15 - 5.35) | 4.27 (3.48 - 5.31) | 0.160 |
| INR | 1.04 (1.00 - 1.10) | 1.04 (0.99 - 1.09) | 1.05 (1.01 - 1.16) | **0.011** |
| ALT (U/L) | 20.0 (13.0 - 35.0) | 20.0 (13.0 - 34.0) | 19.0 (13.5 - 39.0) | 0.798 |
| AST (U/L) | 22.0 (16.0 - 31.0) | 21.0 (16.0 - 31.0) | 23.0 (17.0 - 33.0) | 0.478 |
| ALB (g/L) | 37.6 (33.5 - 41.7) | 38.0 (33.8 - 41.9) | 35.9 (31.4 - 39.9) | **< 0.001** |
| GLOB (g/L) | 30.0 (26.5 - 33.7) | 29.9 (26.3 - 33.6) | 30.5 (27.8 - 33.9) | 0.098 |
| Cr (μmol/L) | 67 (56 - 80) | 67 (56 - 80) | 67 (55 - 86) | 0.990 |
| EGFR (ml/min/1.73m^2) | 93.4 (80.6 - 103.1) | 94.3 (82.1 - 104.4) | 87.1 (68.9 - 95.4) | **< 0.001** |
| GLU (mmol/L) | 5.53 (4.94 - 6.85) | 5.45 (4.92 - 6.82) | 5.72 (5.26 - 7.40) | **0.014** |
| TBIL (μmol/L) | 3.92 (3.31 - 4.67) | 3.98 (3.41 - 4.71) | 3.35 (2.83 - 4.11) | **< 0.001** |
| **Hospital stay-days, median (IQR)** | 23 (14 - 36) | 22 (14 - 34) | 31 (19 - 43) | **< 0.001** |

*p* values were calculated between cardiac and non-cardiac groups by Mann-Whitney U test and chi-square test, as appropriate. Abbreviations: IQR, interquartile range; HP, hypertension; DM, diabetes; COPD, chronic obstructive pulmonary disease; SBP, Systolic blood pressure; DBP, Diastolic blood pressure; SpO_2_, percutaneous oxygen saturation; Hs-TnI, High sensitivity troponin-I; CK-MB, creatine kinase-MB; Myo, myoglobin; NT-proBNP, N terminal pro B type natriuretic peptide; WBC, white blood cell; NEU, neutrophil; NEU%, neutrophil percentage; LYM, lymphocytes; LYM%, lymphocyte percentage; Hs-CRP, high sensitivity C-reactive protein; IL2R, interleukin 2 receptor; IL6, interleukin 6; IL8, interleukin 8; TNFα, tumour necrosis factor α; PLT, platelet; FIB, fibrinogen; INR, international normalized ratio; ALT, alanine aminotransferase; AST, aspartate transaminase; ALB, albumin; GLOB, globulin; Cr, creatinine; EGFR, estimated glomerular filtration rate; GLU, glucose; TBIL, total bilirubin.
