# Supplementary material for: Cardiac Biomarker Levels and Their Prognostic Values in COVID-19 Patients With or Without Concomitant Cardiac Disease
Source: Front Cardiovasc Med. 2021 Jan 20;7:599096. doi: 10.3389/fcvm.2020.599096 (PMC7856675; doi:10.3389/fcvm.2020.599096)
Supplement: Supplementary file 2 [file Table_2.DOCX]

Table S2. Baseline characteristics and on-admission laboratory findings of non-survivors stratified based on concomitant cardiac disease.

| **Characteristics** | **Non-survivors**  **(n = 60)** | **No cardiac disease**  **(n = 43)** | **Cardiac disease**  **(n = 17)** | ***p* value** |
| --- | --- | --- | --- | --- |
| **Age (yrs), median (IQR)** | 73 (67 - 81) | 71 (67 - 81) | 76 (66 - 82) | 0.669 |
| **Male/Female, n** | 42/18 | 30/13 | 12/5 | 0.950 |
| **Comorbidities, n (%)** | | | | |
| History of HP-n (%) | 26 (43.3) | 12 (27.9) | 14 (82.4) | **< 0.001** |
| History of DM -n (%) | 13 (21.7) | 7 (16.3) | 6 (35.3) | 0.107 |
| Chronic liver disease-n (%) | 1 (1.7) | 1 (2.3) | 0 (0) | 0.526 |
| Stroke history-n (%) | 7 (11.7) | 5 (11.6) | 2 (11.8) | 0.988 |
| Chronic kidney disease-n (%) | 6 (10.0) | 2 (4.7) | 4 (23.5) | **0.028** |
| History of COPD-n (%) | 1 (1.7) | 1 (2.3) | 0 (0) | 0.526 |
| Cancer-n (%) | 3 (5.0) | 1 (2.3) | 2 (11.8) | 0.131 |
| **Clinical classifications, n (%)** | | | | |
| Mild cases-n (%) | 0 (0) | 0 (0) | 0 (0) | - |
| Ordinary cases-n (%) | 7 (11.7) | 4 (9.3) | 3 (17.6) | 0.364 |
| Severe cases-n (%) | 7 (11.7) | 3 (7.0) | 4 (23.5) | 0.072 |
| Critical cases-n (%) | 46 (76.7) | 36 (83.7) | 10 (58.8) | **0.040** |
| **Physical examination on admission, median (IQR)** | | | | |
| Temperature (°C) | 36.7 (36.4 - 37.3) | 36.9 (36.4 - 37.4) | 36.6 (36.5 - 37.3) | 0.863 |
| Pulse (/min) | 92 (82 - 102) | 91 (83 - 101) | 92 (79 - 103) | 0.967 |
| Respire (/min) | 21 (20 - 30) | 22 (20 - 30) | 20 (20 - 26) | 0.641 |
| SBP (mmHg) | 130 (119 - 145) | 130 (117 - 152) | 130 (120 - 138) | 0.426 |
| DBP (mmHg) | 78 (70 - 87) | 76 (69 - 87) | 79 (70 - 86) | 0.611 |
| SpO_2_ (%) | 93 (90 - 98) | 92 (87 - 97) | 96 (92 - 98) | **0.047** |
| **Laboratory tests on admission, median (IQR)** | | | | |
| Hs-TnI (pg/mL) | 25.0 (7.5 - 130.5) | 35.3 (5.5 - 296.4) | 16.7 (9.4 - 53.2) | 0.337 |
| CK-MB (ng/mL) | 2.3 (0.8 - 4.3) | 2.9 (1.2 - 4.6) | 1.0 (0.5 - 2.4) | **0.007** |
| Myo (ng/mL) | 151.0 (93.7 - 341.0) | 174.1 (108.4 - 368.6) | 101.9 (76.2 - 197.6) | 0.070 |
| NT-proBNP (pg/mL) | 1011.5 (433.3 - 3004.8) | 1032.0 (359.0 - 3122.0) | 991.0 (676.0 - 2970.5) | 0.583 |
| WBC (10^9/L) | 8.36 (6.09 - 13.25) | 9.01 (6.05 - 14.06) | 7.36 (6.04 - 9.05) | 0.113 |
| NEU (10^9/L) | 7.73 (4.97 - 11.91) | 8.34 (4.90 - 12.57) | 6.67 (5.04 - 8.90) | 0.195 |
| NEU% (%) | 87.6 (79.1 - 91.2) | 88.1 (79.9 - 91.8) | 84.1 (77.1 - 89.7) | 0.149 |
| LYM (10^9/L) | 0.64 (0.43 - 0.95) | 0.56 (0.43 - 1.01) | 0.75 (0.42 - 0.86) | 0.658 |
| LYM% (%) | 7.3 (4.4 - 13.2) | 7.1 (4.3 - 13.2) | 11.2 (4.8 - 14.0) | 0.290 |
| Hs-CRP (mg/L) | 76.3 (37.9 - 142.4) | 89.7 (48.5 - 146.5) | 47.3 (20.4 - 131.0) | **0.049** |
| IL2R (U/mL) | 1043.0 (800.8 - 1482.3) | 1101.0 (827.0 - 1484.0) | 948.0 (657.0 - 1280.5) | 0.337 |
| IL6 (pg/mL) | 56.54 (24.41 - 160.53) | 64.78 (26.56 - 172.10) | 46.98 (15.19 - 72.50) | 0.247 |
| IL8 (pg/mL) | 25.1 (15.7 - 47.8) | 36.7 (20.8 - 52.6) | 13.8 (11.7 - 21.4) | **< 0.001** |
| TNFα (pg/mL) | 12.1 (8.5 - 20.0) | 12.1 (9.3 - 21.4) | 11.3 (7.6 - 15.3) | 0.358 |
| PLT (10^9/L) | 151 (102 - 232) | 122 (92 - 214) | 207 (148 - 275) | **0.021** |
| D-dimer (μg/mL FEU) | 5.08 (1.66 - 21.00) | 5.70 (1.73 - 21.00) | 2.68 (0.78 - 8.89) | 0.059 |
| FIB (g/L) | 4.75 (2.90 - 6.18) | 4.52 (2.20 - 6.74) | 5.35 (4.16 - 5.90) | 0.212 |
| INR | 1.21 (1.11 - 1.32) | 1.22 (1.13 - 1.35) | 1.14 (1.08 - 1.28) | 0.176 |
| ALT (U/L) | 24.5 (17.3 - 43.8) | 30.0 (18.0 - 46.0) | 20.0 (16.5 - 37.5) | 0.549 |
| AST (U/L) | 38.5 (24.0 - 56.8) | 42.0 (24.0 - 59.0) | 33.0 (20.0 - 46.0) | 0.088 |
| ALB (g/L) | 31.6 (28.3 - 34.9) | 31.6 (28.9 - 33.8) | 32.0 (26.3 - 37.4) | 0.948 |
| GLOB (g/L) | 34.8 (30.5 - 38.9) | 34.8 (31.0 - 39.5) | 31.2 (30.4 - 38.5) | 0.555 |
| Cr (μmol/L) | 86 (65 - 119) | 83 (64 - 116) | 94 (68 - 129) | 0.724 |
| EGFR (ml/min/1.73m^2) | 67.4 (47.0 - 92.5) | 66.2 (45.9 - 95.1) | 68.6 (45.9 - 87.8) | 0.948 |
| GLU (mmol/L) | 7.40 (5.99 - 10.63) | 7.18 (5.90 - 10.70) | 7.62 (6.33 - 10.51) | 0.935 |
| TBIL (μmol/L) | 3.28 (2.89 - 3.90) | 3.32 (2.91 - 3.87) | 3.00 (2.19 - 3.97) | 0.329 |
| **Hospital stay-days, median (IQR)** | 14 (11 - 22) | 13 (9 - 18) | 22 (14 - 32) | **0.003** |

*p* values were calculated between cardiac and non-cardiac groups by Mann-Whitney U test and chi-square test, as appropriate. Abbreviations: IQR, interquartile range; HP, hypertension; DM, diabetes; COPD, chronic obstructive pulmonary disease; SBP, Systolic blood pressure; DBP, Diastolic blood pressure; SpO_2_, percutaneous oxygen saturation; Hs-TnI, High sensitivity troponin-I; CK-MB, creatine kinase-MB; Myo, myoglobin; NT-proBNP, N terminal pro B type natriuretic peptide; WBC, white blood cell; NEU, neutrophil; NEU%, neutrophil percentage; LYM, lymphocytes; LYM%, lymphocyte percentage; Hs-CRP, high sensitivity C-reactive protein; IL2R, interleukin 2 receptor; IL6, interleukin 6; IL8, interleukin 8; TNFα, tumor necrosis factor α; PLT, platelet; FIB, fibrinogen; INR, international normalized ratio; ALT, alanine aminotransferase; AST, aspartate transaminase; ALB, albumin; GLOB, globulin; Cr, creatinine; EGFR, estimated glomerular filtration rate; GLU, glucose; TBIL, total bilirubin.
