# Supplementary material for: Cardiac Biomarker Levels and Their Prognostic Values in COVID-19 Patients With or Without Concomitant Cardiac Disease
Source: Front Cardiovasc Med. 2021 Jan 20;7:599096. doi: 10.3389/fcvm.2020.599096 (PMC7856675; doi:10.3389/fcvm.2020.599096)
Supplement: Supplementary file 3 [file Table_3.DOCX]

Table S3. Cardiac biomarker levels in the overall study population, cardiac and non-cardiac patients, stratified by mortality.

|  | **Biomarkers** | **Early stage** | | |  | **Late stage** | | |
| --- | --- | --- | --- | --- | --- | --- | --- | --- |
|  |  | **Alive** | **Died** | ***p* value** |  | **Alive** | **Died** | ***p* value** |
| **All included patients** |  | n = 963 | n = 60 |  |  | n = 963 | n = 60 |  |
|  | Hs-TnI (pg/mL) | 2.8 (1.9 - 7.6) | 25.0 (7.5 - 130.5) | **< 0.001** |  | 2.1 (1.9 - 4.9) | 144.9 (32.7 - 525.0) | **< 0.001** |
|  | CK-MB (ng/mL) | 0.7 (0.5 - 1.1) | 2.3 (0.8 - 4.3) | **< 0.001** |  | 0.6 (0.4 - 0.9) | 4.9 (2.0 - 10.3) | **< 0.001** |
|  | Myo (ng/mL) | 35.0 (26.5 - 55.6) | 151.0 (93.7 - 341.0) | **< 0.001** |  | 29.5 (23.2 - 39.9) | 786.2 (332.3 - 1200.0) | **< 0.001** |
|  | NT-proBNP (pg/mL) | 83.0 (32.0 - 210.0) | 1011.5 (433.3 - 3004.8) | **< 0.001** |  | 64.0 (27.0 - 156.0) | 4871.5 (1443.3 - 11294.5) | **< 0.001** |
| **Patients without history of cardiac disease** |  | n = 854 | n = 43 |  |  | n = 854 | n = 43 |  |
|  | Hs-TnI (pg/mL) | 2.5 (1.9 - 6.5) | 35.3 (5.5 - 296.4) | **< 0.001** |  | 1.9 (1.9 - 4.3) | 156.1 (32.6 - 523.5) | **< 0.001** |
|  | CK-MB (ng/mL) | 0.7 (0.5 - 1.1) | 2.9 (1.2 - 4.6) | **< 0.001** |  | 0.6 (0.4 - 0.8) | 4.5 (2.1 - 10.2) | **< 0.001** |
|  | Myo (ng/mL) | 34.3 (25.7 - 53.2) | 174.1 (108.4 - 368.6) | **< 0.001** |  | 29.1 (23.1 - 38.5) | 869.8 (368.6 - 1200.0) | **< 0.001** |
|  | NT-proBNP (pg/mL) | 72.0 (29.0 - 173.5) | 1032.0 (359.0 - 3122.0) | **< 0.001** |  | 57.0 (25.0 - 134.3) | 4985.0 (1824.0 - 10957.0) | **< 0.001** |
| **Patients with a history of cardiac disease** |  | n = 109 | n = 17 |  |  | n = 109 | n = 17 |  |
|  | Hs-TnI (pg/mL) | 7.6 (3.1 - 19.7) | 16.7 (9.4 - 53.2) | **0.030** |  | 5.3 (2.5 - 13.0) | 107.4 (29.2 - 748.7) | **< 0.001** |
|  | CK-MB (ng/mL) | 1.1 (0.7 - 1.9) | 1.0 (0.5 - 2.4) | 0.673 |  | 0.9 (0.5 - 1.3) | 6.4 (1.7 - 11.6) | **< 0.001** |
|  | Myo (ng/mL) | 47.5 (32.3 - 76.2) | 101.9 (76.2 - 197.6) | **< 0.001** |  | 33.8 (25.0 - 50.2) | 587.3 (211.5 - 1200.0) | **< 0.001** |
|  | NT-proBNP (pg/mL) | 257.0 (94.0 - 1101.0) | 991.0 (676.0 - 2970.5) | **0.001** |  | 169.0 (65.5 - 533.5) | 2596.0 (1207.5 - 12869.5) | **< 0.001** |

*p* values were calculated by Mann-Whitney U test. Abbreviations: Hs-TnI, High sensitivity troponin-I; CK-MB, creatine kinase-MB; Myo, myoglobin; NT-proBNP, N terminal pro B type natriuretic peptide
