# Supplementary material for: Cardiac Biomarker Levels and Their Prognostic Values in COVID-19 Patients With or Without Concomitant Cardiac Disease
Source: Front Cardiovasc Med. 2021 Jan 20;7:599096. doi: 10.3389/fcvm.2020.599096 (PMC7856675; doi:10.3389/fcvm.2020.599096)
Supplement: Supplementary file 4 [file Table_4.DOCX]

Table S4. Clinical characteristics and on-admission laboratory findings of the overall study population patients stratified by mortality.

| **Characteristics** | | **Alive**  **(n = 963)** | **Died**  **(n = 60)** | ***p* value** |
| --- | --- | --- | --- | --- |
| **Age (yrs), median (IQR)** | | 62 (50 - 69) | 73 (67 - 81) | **< 0.001** |
| **Male/Female, n** | | 448/515 | 42/18 | **< 0.001** |
| **Comorbidities, n (%)** | | | | |
| History of cardiac disease-n (%) | | 109 (11.3) | 17 (28.3) | **< 0.001** |
| History of HP-n (%) | | 341 (35.4) | 26 (43.3) | 0.214 |
| History of DM -n (%) | | 156 (16.2) | 13 (21.7) | 0.269 |
| Chronic liver disease-n (%) | | 14 (1.5) | 1 (1.7) | 0.897 |
| Stroke history-n (%) | | 41 (4.3) | 7 (11.7) | **0.008** |
| Chronic kidney disease-n (%) | | 20 (2.1) | 6 (10.0) | **< 0.001** |
| History of COPD-n (%) | | 10 (1.0) | 1 (1.7) | 0.647 |
| Cancer-n (%) | | 35 (3.6) | 3 (5.0) | 0.587 |
| **Clinical classifications, n (%)** | | | | |
| Mild cases-n (%) | 14 (1.5) | | 0 (0) | 0.347 |
| Ordinary cases-n (%) | 735 (76.3) | | 7 (11.7) | **< 0.001** |
| Severe cases-n (%) | 198 (20.6) | | 7 (11.7) | 0.095 |
| Critical cases-n (%) | 16 (1.7) | | 46 (76.7) | **< 0.001** |
| **Physical examination on admission, median (IQR)** | | | | |
| Temperature (°C) | | 36.5 (36.2 - 36.9) | 36.7 (36.4 - 37.3) | **0.010** |
| Pulse (/min) | | 89 (80 - 100) | 92 (82 - 102) | 0.390 |
| Respire (/min) | | 20 (19 - 22) | 21 (20 - 30) | **< 0.001** |
| SBP (mmHg) | | 133 (120 - 145) | 130 (119 - 145) | 0.518 |
| DBP (mmHg) | | 81 (73 - 90) | 78 (70 - 87) | 0.105 |
| SpO_2_ (%) | | 97 (95 - 98) | 93 (90 - 98) | **< 0.001** |
| **Laboratory tests on admission, median (IQR)** | | | | |
| Hs-TnI (pg/mL) | | 2.8 (1.9 - 7.6) | 25.0 (7.5 - 130.5) | **< 0.001** |
| CK-MB (ng/mL) | | 0.7 (0.5 - 1.1) | 2.3 (0.8 - 4.3) | **< 0.001** |
| Myo (ng/mL) | | 35.0 (26.5 - 55.6) | 151.0 (93.7 - 341.0) | **< 0.001** |
| NT-proBNP (pg/mL) | | 83.0 (32.0 - 210.0) | 1011.5 (433.3 - 3004.8) | **< 0.001** |
| WBC (10^9/L) | | 5.90 (4.73 - 7.38) | 8.36 (6.09 - 13.25) | **< 0.001** |
| NEU (10^9/L) | | 3.69 (2.71 - 5.08) | 7.73 (4.97 - 11.91) | **< 0.001** |
| NEU% (%) | | 63.3 (55.5 - 72.1) | 87.6 (79.1 - 91.2) | **< 0.001** |
| LYM (10^9/L) | | 1.36 (0.98 - 1.80) | 0.64 (0.43 - 0.95) | **< 0.001** |
| LYM% (%) | | 25.0 (17.0 - 31.8) | 7.3 (4.4 - 13.2) | **< 0.001** |
| Hs-CRP (mg/L) | | 4.5 (1.2 - 31.6) | 76.3 (37.9 - 142.4) | **< 0.001** |
| IL2R (U/mL) | | 465.0 (309.0 - 716.0) | 1043.0 (800.8 - 1482.3) | **< 0.001** |
| IL6 (pg/mL) | | 3.63 (1.70 - 10.09) | 56.54 (24.41 - 160.53) | **< 0.001** |
| IL8 (pg/mL) | | 9.9 (6.5 - 16.9) | 25.1 (15.7 - 47.8) | **< 0.001** |
| TNFα (pg/mL) | | 8.1 (6.2 - 10.4) | 12.1 (8.5 - 20.0) | **< 0.001** |
| PLT (10^9/L) | | 234 (188 - 302) | 151 (102 - 232) | **< 0.001** |
| D-dimer (μg/mL FEU) | | 0.51 (0.23 - 1.20) | 5.08 (1.66 - 21.00) | **< 0.001** |
| FIB (g/L) | | 4.03 (3.19 - 5.35) | 4.75 (2.90 - 6.18) | 0.130 |
| INR | | 1.04 (1.00 - 1.10) | 1.21 (1.11 - 1.32) | **< 0.001** |
| ALT (U/L) | | 20.0 (13.0 - 35.0) | 24.5 (17.3 - 43.8) | **0.009** |
| AST (U/L) | | 22.0 (16.0 - 31.0) | 38.5 (24.0 - 56.8) | **< 0.001** |
| ALB (g/L) | | 37.6 (33.5 - 41.7) | 31.6 (28.3 - 34.9) | **< 0.001** |
| GLOB (g/L) | | 30.0 (26.5 - 33.7) | 34.8 (30.5 - 38.9) | **< 0.001** |
| Cr (μmol/L) | | 67 (56 - 80) | 86 (65 - 119) | **< 0.001** |
| EGFR (ml/min/1.73m^2) | | 93.4 (80.6 - 103.1) | 67.4 (47.0 - 92.5) | **< 0.001** |
| GLU (mmol/L) | | 5.53 (4.94 - 6.85) | 7.40 (5.99 - 10.63) | **< 0.001** |
| TBIL (μmol/L) | | 3.92 (3.31 - 4.67) | 3.28 (2.89 - 3.90) | **< 0.001** |
| **Hospital stay-days, median (IQR)** | | 23 (14 - 36) | 14 (11 - 22) | **< 0.001** |

*p* values were calculated by Mann-Whitney U test and chi-square test, as appropriate. Abbreviations: IQR, interquartile range; HP, hypertension; DM, diabetes; COPD, chronic obstructive pulmonary disease; SBP, Systolic blood pressure; DBP, Diastolic blood pressure; SpO_2_, percutaneous oxygen saturation; Hs-TnI, High sensitivity troponin-I; CK-MB, creatine kinase-MB; Myo, myoglobin; NT-proBNP, N terminal pro B type natriuretic peptide; WBC, white blood cell; NEU, neutrophil; NEU%, neutrophil percentage; LYM, lymphocytes; LYM%, lymphocyte percentage; Hs-CRP, high sensitivity C-reactive protein; IL2R, interleukin 2 receptor; IL6, interleukin 6; IL8, interleukin 8; TNFα, tumor necrosis factor α; PLT, platelet; FIB, fibrinogen; INR, international normalized ratio; ALT, alanine aminotransferase; AST, aspartate transaminase; ALB, albumin; GLOB, globulin; Cr, creatinine; EGFR, estimated glomerular filtration rate; GLU, glucose; TBIL, total bilirubin.
