# Supplementary material for: Cardiac Biomarker Levels and Their Prognostic Values in COVID-19 Patients With or Without Concomitant Cardiac Disease
Source: Front Cardiovasc Med. 2021 Jan 20;7:599096. doi: 10.3389/fcvm.2020.599096 (PMC7856675; doi:10.3389/fcvm.2020.599096)
Supplement: Supplementary file 5 [file Table_5.DOCX]

Table S5. Clinical characteristics and on-admission laboratory findings of the non-cardiac patients, stratified by mortality.

| **Characteristics** | **Non-cardiac patients**  **(n = 897)** | | **Alive**  **(n = 854)** | **Died**  **(n = 43)** | ***p* value** |
| --- | --- | --- | --- | --- | --- |
| **Age (yrs), median (IQR)** | 61 (50 - 69) | | 60 (49 - 68) | 71 (67 - 81) | **< 0.001** |
| **Male/Female, n** | 427/470 | | 397/457 | 30/13 | **0.003** |
| **Comorbidities, n (%)** | | | | | |
| History of HP-n (%) | 273 (30.4) | | 261 (30.6) | 12 (27.9) | 0.712 |
| History of DM -n (%) | 131 (14.6) | | 124 (14.5) | 7 (16.3) | 0.750 |
| Chronic liver disease-n (%) | 15 (1.7) | | 14 (1.6) | 1 (2.3) | 0.732 |
| Stroke history-n (%) | 37 (4.1) | | 32 (3.7) | 5 (11.6) | **0.011** |
| Chronic kidney disease-n (%) | 17 (1.9) | | 15 (1.8) | 2 (4.7) | 0.174 |
| History of COPD-n (%) | 8 (0.9) | | 7 (0.8) | 1 (2.3) | 0.305 |
| Cancer-n (%) | 28 (3.1) | | 27 (3.2) | 1 (2.3) | 0.758 |
| **Clinical classifications, n (%)** | | | | | |
| Mild cases-n (%) | 14 (1.6) | 14 (1.6) | | 0 (0) | 0.397 |
| Ordinary cases-n (%) | 665 (74.1) | 661 (77.4) | | 4 (9.3) | **< 0.001** |
| Severe cases-n (%) | 171 (19.1) | 168 (19.7) | | 3 (7.0) | **0.039** |
| Critical cases-n (%) | 47 (5.2) | 11 (1.3) | | 36 (83.7) | **< 0.001** |
| **Physical examination on admission, median (IQR)** | | | | | |
| Temperature (°C) | 36.5 (36.2 - 36.9) | | 36.5 (36.2 - 36.8) | 36.9 (36.4 - 37.4) | **0.025** |
| Pulse (/min) | 90 (80 - 100) | | 90 (80 - 100) | 91 (83 - 101) | 0.530 |
| Respire (/min) | 20 (19 - 22) | | 20 (19 - 22) | 22 (20 - 30) | **0.001** |
| SBP (mmHg) | 132 (120 - 145) | | 132 (120 - 145) | 130 (117 - 152) | 0.886 |
| DBP (mmHg) | 81 (72 - 90) | | 81 (73 - 90) | 76 (69 - 87) | 0.096 |
| SpO_2_ (%) | 97 (95 - 98) | | 97 (95 - 98) | 92 (87 - 97) | **< 0.001** |
| **Laboratory tests on admission, median (IQR)** | | | | | |
| Hs-TnI (pg/mL) | 2.7 (1.9 - 7.5) | | 2.5 (1.9 - 6.5) | 35.3 (5.5 - 296.4) | **< 0.001** |
| CK-MB (ng/mL) | 0.7 (0.5 - 1.1) | | 0.7 (0.5 - 1.1) | 2.9 (1.2 - 4.6) | **< 0.001** |
| Myo (ng/mL) | 34.9 (26.0 - 57.4) | | 34.3 (25.7 - 53.2) | 174.1 (108.4 - 368.6) | **< 0.001** |
| NT-proBNP (pg/mL) | 80.0 (30.0 - 198.5) | | 72.0 (29.0 - 173.5) | 1032.0 (359.0 - 3122.0) | **< 0.001** |
| WBC (10^9/L) | 5.91 (4.73 - 7.44) | | 5.86 (4.67 - 7.34) | 9.01 (6.05 - 14.06) | **< 0.001** |
| NEU (10^9/L) | 3.69 (2.71 - 5.13) | | 3.61 (2.67 - 4.96) | 8.34 (4.90 - 12.57) | **< 0.001** |
| NEU% (%) | 63.3 (55.6 - 72.9) | | 62.7 (55.1 - 71.6) | 88.1 (79.9 - 91.8) | **< 0.001** |
| LYM (10^9/L) | 1.35 (0.97 - 1.81) | | 1.38 (1.01 - 1.83) | 0.56 (0.43 - 1.01) | **< 0.001** |
| LYM% (%) | 25.1 (16.6 - 32.0) | | 25.8 (17.8 - 32.2) | 7.1 (4.3 - 13.2) | **< 0.001** |
| Hs-CRP (mg/L) | 4.6 (1.1 - 35.2) | | 4.0 (1.0 - 27.4) | 89.7 (48.5 - 146.5) | **< 0.001** |
| IL2R (U/mL) | 462.0 (306.0 - 724.5) | | 448.5 (299.5 - 693.3) | 1101.0 (827.0 - 1484.0) | **< 0.001** |
| IL6 (pg/mL) | 3.59 (1.66 - 10.60) | | 3.35 (1.59 - 8.72) | 64.78 (26.56 - 172.10) | **< 0.001** |
| IL8 (pg/mL) | 9.9 (6.5 - 18.0) | | 9.4 (6.4 - 16.1) | 36.7 (20.8 - 52.6) | **< 0.001** |
| TNFα (pg/mL) | 8.1 (6.2 - 10.4) | | 8.0 (6.1 - 10.2) | 12.1 (9.3 - 21.4) | **< 0.001** |
| PLT (10^9/L) | 234 (184 - 300) | | 236 (191 - 303) | 122 (92 - 214) | **< 0.001** |
| D-dimer (μg/mL FEU) | 0.51 (0.22 - 1.27) | | 0.49 (0.22 - 1.11) | 5.70 (1.73 - 21.00) | **< 0.001** |
| FIB (g/L) | 4.02 (3.14 - 5.39) | | 4.01 (3.15 - 5.35) | 4.52 (2.20 - 6.74) | 0.707 |
| INR | 1.05 (1.00 - 1.10) | | 1.04 (0.99 - 1.09) | 1.22 (1.13 - 1.35) | **< 0.001** |
| ALT (U/L) | 21.0 (13.0 - 35.0) | | 20.0 (13.0 - 34.0) | 30.0 (18.0 - 46.0) | **0.012** |
| AST (U/L) | 22.0 (16.0 - 32.0) | | 21.0 (16.0 - 31.0) | 42.0 (24.0 - 59.0) | **< 0.001** |
| ALB (g/L) | 37.6 (33.4 - 41.7) | | 38.0 (33.8 - 41.9) | 31.6 (28.9 - 33.8) | **< 0.001** |
| GLOB (g/L) | 30.0 (26.3 - 34.0) | | 29.9 (26.3 - 33.6) | 34.8 (31.0 - 39.5) | **< 0.001** |
| Cr (μmol/L) | 67 (57 - 80) | | 67 (56 - 80) | 83 (64 - 116) | **< 0.001** |
| EGFR (ml/min/1.73m^2) | 93.6 (80.6 - 103.9) | | 94.3 (82.1 - 104.4) | 66.2 (45.9 - 95.1) | **< 0.001** |
| GLU (mmol/L) | 5.53 (4.94 - 6.98) | | 5.45 (4.92 - 6.82) | 7.18 (5.90 - 10.70) | **< 0.001** |
| TBIL (μmol/L) | 3.93 (3.35 - 4.67) | | 3.98 (3.41 - 4.71) | 3.32 (2.91 - 3.87) | **< 0.001** |
| **Hospital stay-days, median (IQR)** | 22 (13 - 34) | | 22 (14 - 34) | 13 (9 - 18) | **< 0.001** |

*p* values were calculated between alive and died groups by Mann-Whitney U test and chi-square test, as appropriate. Abbreviations: IQR, interquartile range; HP, hypertension; DM, diabetes; COPD, chronic obstructive pulmonary disease; SBP, Systolic blood pressure; DBP, Diastolic blood pressure; SpO_2_, percutaneous oxygen saturation; Hs-TnI, High sensitivity troponin-I; CK-MB, creatine kinase-MB; Myo, myoglobin; NT-proBNP, N terminal pro B type natriuretic peptide; WBC, white blood cell; NEU, neutrophil; NEU%, neutrophil percentage; LYM, lymphocytes; LYM%, lymphocyte percentage; Hs-CRP, high sensitivity C-reactive protein; IL2R, interleukin 2 receptor; IL6, interleukin 6; IL8, interleukin 8; TNFα, tumor necrosis factor α; PLT, platelet; FIB, fibrinogen; INR, international normalized ratio; ALT, alanine aminotransferase; AST, aspartate transaminase; ALB, albumin; GLOB, globulin; Cr, creatinine; EGFR, estimated glomerular filtration rate; GLU, glucose; TBIL, total bilirubin.
