# Supplementary material for: Cardiac Biomarker Levels and Their Prognostic Values in COVID-19 Patients With or Without Concomitant Cardiac Disease
Source: Front Cardiovasc Med. 2021 Jan 20;7:599096. doi: 10.3389/fcvm.2020.599096 (PMC7856675; doi:10.3389/fcvm.2020.599096)
Supplement: Supplementary file 6 [file Table_6.DOCX]

Table S6. Clinical characteristics and on-admission laboratory findings of the patients with cardiac disease, stratified by mortality.

| **Characteristics** | **Cardiac patients**  **(n = 126)** | **Alive**  **(n = 109)** | **Died**  **(n = 17)** | ***p* value** |
| --- | --- | --- | --- | --- |
| **Age (yrs), median (IQR)** | 72 (64 - 80) | 71 (64 - 80) | 76 (66 - 82) | 0.395 |
| **Male/Female, n** | 63/63 | 51/58 | 12/5 | 0.068 |
| **Comorbidities, n (%)** | | | | |
| History of HP-n (%) | 94 (74.6) | 80 (73.4) | 14 (82.4) | 0.430 |
| History of DM -n (%) | 38 (30.2) | 32 (29.4) | 6 (35.3) | 0.620 |
| Stroke history-n (%) | 11 (8.7) | 9 (8.3) | 2 (11.8) | 0.634 |
| Chronic kidney disease-n (%) | 9 (7.1) | 5 (4.6) | 4 (23.5) | **0.005** |
| History of COPD-n (%) | 3 (2.4) | 3 (2.8) | 0 (0) | 0.489 |
| Cancer-n (%) | 10 (7.9) | 8 (7.3) | 2 (11.8) | 0.530 |
| **Clinical classifications, n (%)** | | | | |
| Mild cases-n (%) | 0 (0) | 0 (0) | 0 (0) | - |
| Ordinary cases-n (%) | 77 (61.1) | 74 (67.9) | 3 (17.6) | **< 0.001** |
| Severe cases-n (%) | 34 (27.0) | 30 (27.5) | 4 (23.5) | 0.730 |
| Critical cases-n (%) | 15 (11.9) | 5 (4.6) | 10 (58.8) | **< 0.001** |
| **Physical examination on admission, median (IQR)** | | | | |
| Temperature (°C) | 36.5 (36.3 - 37.0) | 36.5 (36.3 - 37.0) | 36.6 (36.5 - 37.3) | 0.196 |
| Pulse (/min) | 87 (78 - 100) | 87 (76 - 100) | 92 (79 - 103) | 0.319 |
| Respire (/min) | 20 (19 - 24) | 20 (19 - 24) | 20 (20 - 26) | 0.272 |
| SBP (mmHg) | 137 (121 - 148) | 138 (122 - 150) | 130 (120 - 138) | **0.025** |
| DBP (mmHg) | 79 (72 - 88) | 78 (72 - 88) | 79 (70 - 86) | 0.932 |
| SpO_2_ (%) | 97 (95 - 98) | 97 (95 - 98) | 96 (92 - 98) | 0.555 |
| **Laboratory tests on admission, median (IQR)** | | | | |
| Hs-TnI (pg/mL) | 8.7 (3.3 - 20.9) | 7.6 (3.1 - 19.7) | 16.7 (9.4 - 53.2) | **0.030** |
| CK-MB (ng/mL) | 1.1 (0.7 - 1.9) | 1.1 (0.7 - 1.9) | 1.0 (0.5 - 2.4) | 0.673 |
| Myo (ng/mL) | 53.7 (33.6 - 93.4) | 47.5 (32.3 - 76.2) | 101.9 (76.2 - 197.6) | **< 0.001** |
| NT-proBNP (pg/mL) | 352.5 (109.5 - 1291.5) | 257.0 (94.0 - 1101.0) | 991.0 (676.0 - 2970.5) | **0.001** |
| WBC (10^9/L) | 6.38 (5.13 - 8.23) | 6.09 (5.08 - 7.94) | 7.36 (6.04 - 9.05) | 0.092 |
| NEU (10^9/L) | 4.61 (3.22 - 6.19) | 4.25 (3.11 - 5.77) | 6.67 (5.04 - 8.90) | **0.003** |
| NEU% (%) | 70.8 (61.6 - 82.2) | 69.3 (60.0 - 78.8) | 84.1 (77.1 - 89.7) | **< 0.001** |
| LYM (10^9/L) | 1.09 (0.71 - 1.55) | 1.13 (0.77 - 1.59) | 0.75 (0.42 - 0.86) | **0.003** |
| LYM% (%) | 17.5 (10.6 - 26.1) | 18.9 (11.7 - 26.5) | 11.2 (4.8 - 14.0) | **0.002** |
| Hs-CRP (mg/L) | 18.8 (3.4 - 49.4) | 13.5 (2.7 - 44.7) | 47.3 (20.4 - 131.0) | **0.003** |
| IL2R (U/mL) | 676.5 (453.5 - 1032.8) | 651.0 (436.5 - 995.0) | 948.0 (657.0 - 1280.5) | **0.014** |
| IL6 (pg/mL) | 10.56 (3.34 - 25.27) | 5.61 (3.13 - 20.43) | 46.98 (15.19 - 72.50) | **< 0.001** |
| IL8 (pg/mL) | 13.6 (8.6 - 22.3) | 13.1 (8.4 - 22.5) | 13.8 (11.7 - 21.4) | 0.473 |
| TNFα (pg/mL) | 9.7 (7.3 - 12.5) | 9.6 (7.3 - 12.2) | 11.3 (7.6 - 15.3) | 0.202 |
| PLT (10^9/L) | 218 (165 - 294) | 220 (170 - 300) | 207 (148 - 275) | 0.275 |
| D-dimer (μg/mL FEU) | 0.81 (0.42 - 2.50) | 0.78 (0.38 - 2.30) | 2.68 (0.78 - 8.89) | **0.002** |
| FIB (g/L) | 4.36 (3.51 - 5.45) | 4.27 (3.48 - 5.31) | 5.35 (4.16 - 5.90) | **0.050** |
| INR | 1.07 (1.01 - 1.17) | 1.05 (1.01 - 1.16) | 1.14 (1.08 - 1.28) | **0.012** |
| ALT (U/L) | 19.0 (14.0 - 38.5) | 19.0 (13.5 - 39.0) | 20.0 (16.5 - 37.5) | 0.289 |
| AST (U/L) | 24.0 (17.0 - 34.0) | 23.0 (17.0 - 33.0) | 33.0 (20.0 - 46.0) | 0.062 |
| ALB (g/L) | 35.6 (30.6 - 39.5) | 35.9 (31.4 - 39.9) | 32.0 (26.3 - 37.4) | **0.035** |
| GLOB (g/L) | 30.7 (28.2 - 35.2) | 30.5 (27.8 - 33.9) | 31.2 (30.4 - 38.5) | **0.031** |
| Cr (μmol/L) | 69 (55 - 89) | 67 (55 - 86) | 94 (68 - 129) | **0.005** |
| EGFR (ml/min/1.73m^2) | 84.9 (67.3 - 94.4) | 87.1 (68.9 - 95.4) | 68.6 (45.9 - 87.8) | **0.030** |
| GLU (mmol/L) | 5.96 (5.29 - 7.62) | 5.72 (5.26 - 7.40) | 7.62 (6.33 - 10.51) | **0.002** |
| TBIL (μmol/L) | 3.34 (2.68 - 4.05) | 3.35 (2.83 - 4.11) | 3.00 (2.19 - 3.97) | 0.324 |
| **Hospital stay-days, median (IQR)** | 30 (18 - 42) | 31 (19 - 43) | 22 (14 - 32) | 0.092 |

*p* values were calculated between alive and died groups by Mann-Whitney U test and chi-square test, as appropriate. Abbreviations: IQR, interquartile range; HP, hypertension; DM, diabetes; COPD, chronic obstructive pulmonary disease; SBP, Systolic blood pressure; DBP, Diastolic blood pressure; SpO_2_, percutaneous oxygen saturation; Hs-TnI, High sensitivity troponin-I; CK-MB, creatine kinase-MB; Myo, myoglobin; NT-proBNP, N terminal pro B type natriuretic peptide; WBC, white blood cell; NEU, neutrophil; NEU%, neutrophil percentage; LYM, lymphocytes; LYM%, lymphocyte percentage; Hs-CRP, high sensitivity C-reactive protein; IL2R, interleukin 2 receptor; IL6, interleukin 6; IL8, interleukin 8; TNFα, tumor necrosis factor α; PLT, platelet; FIB, fibrinogen; INR, international normalized ratio; ALT, alanine aminotransferase; AST, aspartate transaminase; ALB, albumin; GLOB, globulin; Cr, creatinine; EGFR, estimated glomerular filtration rate; GLU, glucose; TBIL, total bilirubin.
