# Supplementary material for: Cardiac Biomarker Levels and Their Prognostic Values in COVID-19 Patients With or Without Concomitant Cardiac Disease
Source: Front Cardiovasc Med. 2021 Jan 20;7:599096. doi: 10.3389/fcvm.2020.599096 (PMC7856675; doi:10.3389/fcvm.2020.599096)
Supplement: Supplementary file 7 [file Table_7.DOCX]

Table S7. Cardiac biomarker levels at the early and the late stages in the overall study population, survivors and non-survivors, stratified by concomitant cardiac disease.

|  | **Biomarkers** | **Non-cardiac patients** | | |  | **Cardiac patients** | | |
| --- | --- | --- | --- | --- | --- | --- | --- | --- |
|  |  | **Early stage** | **Late stage** | ***p* value** |  | **Early stage** | **Late stage** | ***p* value** |
| **All included patients** |  | **n = 897** | |  |  | **n = 126** | |  |
|  | Hs-TnI (pg/mL) | 2.7 (1.9 - 7.5) | 2.1 (1.9 - 4.9) | **< 0.001** |  | 8.7 (3.3 - 20.9) | 6.9 (2.7 - 20.9) | 0.126 |
|  | CK-MB (ng/mL) | 0.7 (0.5 - 1.1) | 0.6 (0.4 - 0.9) | **< 0.001** |  | 1.1 (0.7 - 1.9) | 1.0 (0.5 - 1.6) | **0.021** |
|  | Myo (ng/mL) | 34.9 (26.0 - 57.4) | 29.8 (23.4 - 41.2) | **< 0.001** |  | 53.7 (33.6 - 93.4) | 38.1 (27.4 - 60.2) | **0.008** |
|  | NT-proBNP (pg/mL) | 80.0 (30.0 - 198.5) | 63.0 (26.0 - 156.5) | **< 0.001** |  | 352.5 (109.5 - 1291.5) | 267.5 (82.3 - 1198.8) | 0.455 |
| **Alive** |  | **n = 854** | |  |  | **n = 109** | |  |
|  | Hs-TnI (pg/mL) | 2.5 (1.9 - 6.5) | 1.9 (1.9 - 4.3) | **< 0.001** |  | 7.6 (3.1 - 19.7) | 5.3 (2.5 - 13.0) | **< 0.001** |
|  | CK-MB (ng/mL) | 0.7 (0.5 - 1.1) | 0.6 (0.4 - 0.8) | **< 0.001** |  | 1.1 (0.7 - 1.9) | 0.9 (0.5 - 1.3) | **0.002** |
|  | Myo (ng/mL) | 34.3 (25.7 - 53.2) | 29.1 (23.1 - 38.5) | **< 0.001** |  | 47.5 (32.3 - 76.2) | 33.8 (25.0 - 50.2) | **< 0.001** |
|  | NT-proBNP (pg/mL) | 72.0 (29.0 - 173.5) | 57.0 (25.0 - 134.3) | **< 0.001** |  | 257.0 (94.0 - 1101.0) | 169.0 (65.5 - 533.5) | **0.007** |
| **Died** |  | **n = 43** | |  |  | **n = 17** | |  |
|  | Hs-TnI (pg/mL) | 35.3 (5.5 - 296.4) | 156.1 (32.6 - 523.5) | 0.256 |  | 16.7 (9.4 - 53.2) | 107.4 (29.2 - 748.7) | **0.001** |
|  | CK-MB (ng/mL) | 2.9 (1.2 - 4.6) | 4.5 (2.1 - 10.2) | **0.033** |  | 1.0 (0.5 - 2.4) | 6.4 (1.7 - 11.6) | **0.002** |
|  | Myo (ng/mL) | 174.1 (108.4 - 368.6) | 869.8 (368.6 - 1200.0) | **< 0.001** |  | 101.9 (76.2 - 197.6) | 587.3 (211.5 - 1200.0) | **0.003** |
|  | NT-proBNP (pg/mL) | 1032.0 (359.0 - 3122.0) | 4985.0 (1824.0 - 10957.0) | **< 0.001** |  | 991.0 (676.0 - 2970.5) | 2596.0 (1207.5 - 12869.5) | **0.003** |

*p* values were calculated between the early-stage and the late-stage groups by Wilcoxon signed-ranks test (two-tailed). Abbreviations: Hs-TnI, High sensitivity troponin-I; CK-MB, creatine kinase-MB; Myo, myoglobin; NT-proBNP, N terminal pro B type natriuretic peptide
