# Supplementary material for: Cardiac Biomarker Levels and Their Prognostic Values in COVID-19 Patients With or Without Concomitant Cardiac Disease
Source: Front Cardiovasc Med. 2021 Jan 20;7:599096. doi: 10.3389/fcvm.2020.599096 (PMC7856675; doi:10.3389/fcvm.2020.599096)
Supplement: Supplementary file 8 [file Table_8.DOCX]

Table S8. Univariate Cox regression analyses showing the association between variables and the risk of in-hospital mortality in overall study population (n=1023).

|  |  |  |  |  |  | 95.0% CI for Exp(B) | |  |
| --- | --- | --- | --- | --- | --- | --- | --- | --- |
| Variables | B | SE | Wald χ^2^ | df | Exp(B) | Lower | Upper | Sig. |
| Age | 0.066 | 0.012 | 30.567 | 1 | 1.068 | 1.043 | 1.093 | **< 0.001** |
| Male vs. Female | 0.950 | 0.282 | 11.374 | 1 | 2.587 | 1.489 | 4.493 | **0.001** |
| History of cardiac disease | 0.846 | 0.288 | 8.654 | 1 | 2.331 | 1.326 | 4.096 | **0.003** |
| History of HP | 0.154 | 0.261 | 0.347 | 1 | 1.166 | 0.699 | 1.946 | 0.556 |
| History of DM | 0.169 | 0.314 | 0.291 | 1 | 1.184 | 0.640 | 2.191 | 0.590 |
| Chronic liver disease | 0.146 | 1.011 | 0.021 | 1 | 1.157 | 0.159 | 8.397 | 0.885 |
| Stroke history | 0.863 | 0.403 | 4.592 | 1 | 2.370 | 1.076 | 5.218 | **0.032** |
| Chronic kidney disease | 1.228 | 0.431 | 8.106 | 1 | 3.415 | 1.466 | 7.953 | **0.004** |
| History of COPD | 0.139 | 1.012 | 0.019 | 1 | 1.149 | 0.158 | 8.343 | 0.891 |
| Cancer | 0.199 | 0.593 | 0.112 | 1 | 1.220 | 0.381 | 3.904 | 0.738 |
| Temperature | 0.312 | 0.167 | 3.473 | 1 | 1.366 | 0.984 | 1.895 | 0.062 |
| Pulse | 0.008 | 0.008 | 0.861 | 1 | 1.008 | 0.991 | 1.025 | 0.354 |
| Respire rate | 0.077 | 0.022 | 12.283 | 1 | 1.080 | 1.034 | 1.127 | **< 0.001** |
| SBP | -0.002 | 0.007 | 0.064 | 1 | 0.998 | 0.985 | 1.012 | 0.800 |
| DBP | -0.007 | 0.011 | 0.493 | 1 | 0.993 | 0.972 | 1.013 | 0.483 |
| SpO_2_ | -0.054 | 0.011 | 26.632 | 1 | 0.947 | 0.928 | 0.967 | **< 0.001** |
| Hs-TnI | 0.000 | 0.000 | 22.492 | 1 | 1.000 | 1.000 | 1.000 | **< 0.001** |
| CK-MB | 0.074 | 0.012 | 39.335 | 1 | 1.077 | 1.052 | 1.103 | **< 0.001** |
| Myo | 0.003 | 0.000 | 95.321 | 1 | 1.003 | 1.002 | 1.003 | **< 0.001** |
| NT-proBNP | 0.000 | 0.000 | 46.225 | 1 | 1.000 | 1.000 | 1.000 | **< 0.001** |
| WBC | 0.126 | 0.016 | 58.611 | 1 | 1.135 | 1.098 | 1.172 | **< 0.001** |
| NEU | 0.164 | 0.015 | 113.585 | 1 | 1.179 | 1.144 | 1.215 | **< 0.001** |
| LYM | -2.245 | 0.339 | 43.927 | 1 | 0.106 | 0.055 | 0.206 | **< 0.001** |
| Hs-CRP | 0.014 | 0.001 | 96.872 | 1 | 1.015 | 1.012 | 1.017 | **< 0.001** |
| IL2R | 0.001 | 0.000 | 76.195 | 1 | 1.001 | 1.000 | 1.001 | **< 0.001** |
| IL6 | 0.002 | 0.000 | 69.319 | 1 | 1.002 | 1.001 | 1.002 | **< 0.001** |
| IL8 | 0.007 | 0.001 | 29.028 | 1 | 1.007 | 1.005 | 1.010 | **< 0.001** |
| TNFα | 0.080 | 0.011 | 52.204 | 1 | 1.084 | 1.060 | 1.107 | **< 0.001** |
| PLT | -0.010 | 0.002 | 38.233 | 1 | 0.990 | 0.987 | 0.993 | **< 0.001** |
| D-dimer | 0.061 | 0.007 | 66.253 | 1 | 1.062 | 1.047 | 1.078 | **< 0.001** |
| FIB | 0.024 | 0.082 | 0.086 | 1 | 1.024 | 0.872 | 1.203 | 0.770 |
| INR | 1.067 | 0.147 | 52.790 | 1 | 2.907 | 2.180 | 3.877 | **< 0.001** |
| ALT | 0.002 | 0.000 | 18.970 | 1 | 1.002 | 1.001 | 1.002 | **< 0.001** |
| AST | 0.001 | 0.000 | 19.193 | 1 | 1.001 | 1.001 | 1.002 | **< 0.001** |
| ALB | 0.065 | 0.015 | 18.395 | 1 | 1.067 | 1.036 | 1.099 | **< 0.001** |
| GLOB | -0.149 | 0.026 | 32.803 | 1 | 0.862 | 0.819 | 0.907 | **< 0.001** |
| Cr | 0.004 | 0.001 | 30.800 | 1 | 1.004 | 1.003 | 1.005 | **< 0.001** |
| EGFR | -0.033 | 0.005 | 54.626 | 1 | 0.967 | 0.959 | 0.976 | **< 0.001** |
| GLU | 0.099 | 0.019 | 26.314 | 1 | 1.104 | 1.063 | 1.147 | **< 0.001** |
| TBIL | -0.460 | 0.137 | 11.220 | 1 | 0.631 | 0.482 | 0.826 | **0.001** |

Abbreviations: HP, hypertension; DM, diabetes; COPD, chronic obstructive pulmonary disease; SBP, Systolic blood pressure; DBP, Diastolic blood pressure; SpO_2_, percutaneous oxygen saturation; Hs-TnI, High sensitivity troponin-I; CK-MB, creatine kinase-MB; Myo, myoglobin; NT-proBNP, N terminal pro B type natriuretic peptide; WBC, white blood cell; NEU, neutrophil; LYM, lymphocytes; Hs-CRP, high sensitivity C-reactive protein; IL2R, interleukin 2 receptor; IL6, interleukin 6; IL8, interleukin 8; TNFα, tumor necrosis factor α; PLT, platelet; FIB, fibrinogen; INR, international normalized ratio; ALT, alanine aminotransferase; AST, aspartate transaminase; ALB, albumin; GLOB, globulin; Cr, creatinine; EGFR, estimated glomerular filtration rate; GLU, glucose; TBIL, total bilirubin; Sig, significance; CI, confidence interval.
