# Supplementary figures and images for: Cardiac Biomarker Levels and Their Prognostic Values in COVID-19 Patients With or Without Concomitant Cardiac Disease
Source: Front Cardiovasc Med. 2021 Jan 20;7:599096. doi: 10.3389/fcvm.2020.599096 (PMC7856675; doi:10.3389/fcvm.2020.599096)

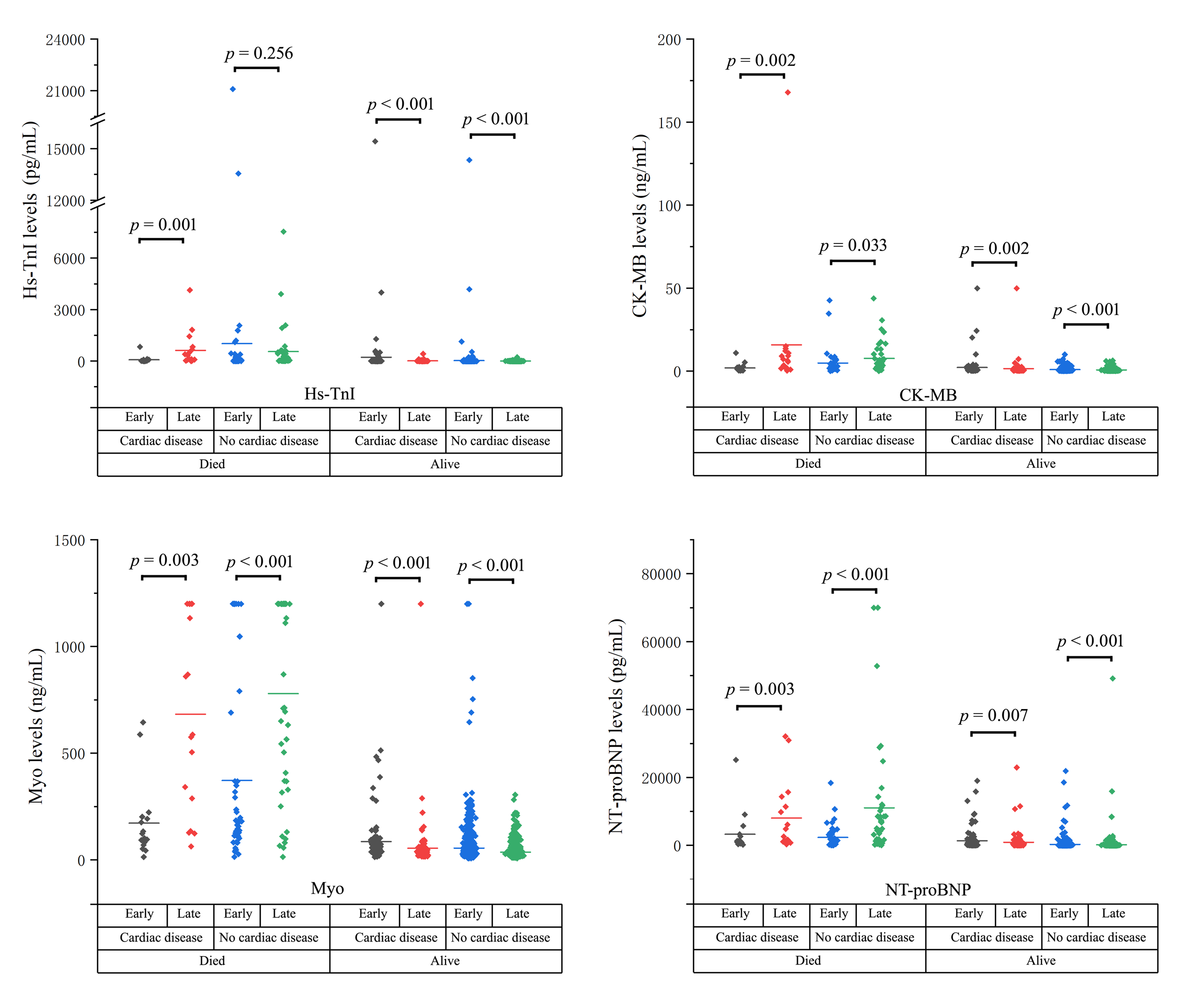

Supplement: Supplementary file 9 [file Image_1.TIF]
